# Rapid Atomic Structure Prediction of Multimetallic Nanoparticles with Physics-Based Machine Learning

**DOI:** 10.1021/acsomega.5c04082

**Published:** 2025-07-14

**Authors:** Bassel Alkhatib, Maya Salem, Klaertje Kiyora Hesselink, Giannis Mpourmpakis

**Affiliations:** † Department of Chemical and Petroleum Engineering, 6614 University of Pittsburgh, Pittsburgh, Pennsylvania 15261, United States

## Abstract

Metal nanoparticles (NPs) find tremendous application in various fields, including catalysis, biomedicine, and electronics, due to their unique physicochemical properties arising from their morphology (i.e., size and shape) and composition. The chemical ordering of NPs, consisting of more than one metal, is crucial for optimizing their application performance, including stability. Traditionally, Density Functional Theory (DFT) has been used to investigate NP stability, but it is computationally expensive, limited to small systems, and cannot be applied to multimetallic NPs, which have enormous materials space. To address this limitation, recent efforts coupled a physics-based model (Bond-Centric Model) with a developed genetic algorithm to optimize the chemical ordering of NPs, leading to minimum (most exothermic) cohesive energies. Central to this approach is the calculation of weighting factors that scale the monometallic bond strength to describe that of the bimetallic bond. Herein, we perform a critical analysis and set some rules on how to apply these methods for rapid and accurate chemical ordering prediction of multimetallic NPs. Specifically, we optimized the chemical ordering of 2869-atom cuboctahedron NPs across 15 different bimetallic combinations and at varying metal compositions. In comparison with both experimental and computational results, our findings indicate that the use of small metal dimers for the calculation of the weighting factors leads to accurate and computationally efficient chemical ordering and stability predictions for a wide range of NP compositions. We further extended our investigation to 6 trimetallic NPs with a tremendously large materials space, testing our model’s capability to predict chemical ordering patterns in multimetallic systems and demonstrating its power as a rapid and accurate computational method. This methodology can facilitate the design of thermodynamically stable multimetallic NPs and predict the distribution of different metal atoms from the core to the surface, which is central to any nanotechnological application.

## Graphical abstract

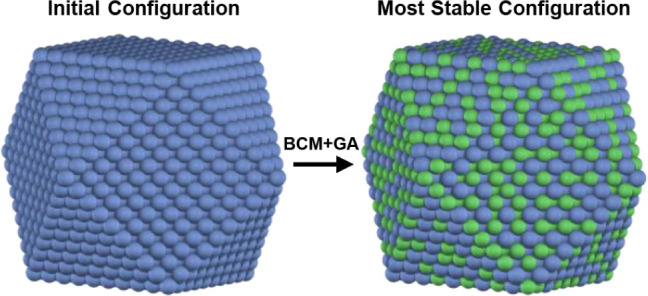

## Introduction

The fields of catalysis^1^ and biomedicine^2^ have seen significant advancements with the emergence of metal NPs. This is mainly attributed to their unique properties, including adsorption and electronic behavior, which stem from their small size and large surface area compared to their bulk.^3,4^ These properties are influenced by factors such as NP morphology, as depicted in their size and shape. Among NPs, multimetallic NPs, composed of two or more metals, exhibit further tunable properties based on their metal composition and chemical ordering.^5–9^ Hence, understanding the chemical ordering in NPs as a function of morphology is crucial for gaining insights into their application performance, including their stability.

Recent studies have highlighted the impact of chemical ordering on the stability of NPs. Molecular dynamics (MD) simulations conducted on Pt–Co NPs elucidated how different chemical orderings influence thermal stability, with ordered intermetallic Pt–Co NPs exhibiting higher melting points and superior stability compared to disordered counterparts. This is attributed to the lower potential energy and greater structural stability of the ordered intermetallics, which result from a more favorable arrangement of Pt–Co bonds. Specifically, the number of Pt–Co bonds is significantly higher in ordered intermetallics, contributing to their enhanced stability. In contrast, disordered alloys show higher diffusivity and mobility of atoms, which facilitates melting at lower temperatures.^10^ Similarly, the stability of ordered PtCu_3_ is higher compared to disordered counterparts due to its enhanced structural integrity, which helps maintain the NP morphology during electrochemical cycling. The ordered arrangement significantly reduces the leaching of copper during reactions, thereby mitigating performance degradation over time, making these PtCu_3_ NPs more effective for applications like oxygen reduction in fuel cells.^11^

Apart from stability, the chemical ordering of the NP affects the catalytic properties (i.e., selectivity and activity). For instance, the arrangement of Pd atoms on the Au surface can significantly enhance the electrocatalytic efficiency in CO_2_ reduction. Dispersing Pd atoms onto the Au surface lowered the barrier required for activating CO_2_ and mitigated the negative impact of strongly binding CO intermediates.^12^ This optimal arrangement can lead to increased rates of product formation and facilitates a more balanced and efficient catalytic process compared to using their monometallic counterparts.^8^ Similarly, the chemical ordering in AuCu NPs significantly affects the selectivity to CO in the CO_2_ electrochemical reduction. Disordered AuCu NPs primarily generate hydrogen gas, while ordered AuCu NPs can achieve a faradaic efficiency for CO production reaching approximately 80%. This selectivity change is attributed to the formation of compressively strained three-atom-thick gold overlayers over the intermetallic core, which results from the disorder-to-order transformation.^13^ These findings indicate the pivotal role of chemical ordering in tailoring the catalytic performance of the NPs.

A comprehensive understanding of NPs and their stability in relation to the factors mentioned previously is essential for tailoring NPs to specific applications. While experimental materials characterization can address NP stability, it is often prohibitively expensive and time-consuming, limiting its accessibility.^14,15^ For instance, the detailed atomic ordering of multimetallic NPs can be characterized experimentally using several advanced techniques, often in combination with each other, including X-ray Photoelectron Spectroscopy (XPS), Auger Electron Spectroscopy (AES), Secondary Ion Mass Spectrometry (SIMS), Low Energy Ion Scattering (LEIS), Scanning Tunneling Microscopy (STM), and Atomic Force Microscopy (AFM).^16,17^ XPS is particularly valuable for providing qualitative information about the near-surface region, allowing for the identification of elements and their oxidation states; however, it requires vacuum conditions and cannot analyze individual particles, necessitating the collection of multiple particles for effective analysis.^16^ AES complements this by enabling the characterization of individual NPs, but it also requires vacuum and is prone to beam damage, especially with sensitive materials.^16^ SIMS offers molecular insights and can detect trace elements on surfaces, yet it typically cannot analyze individual particles and demands meticulous sample preparation.^16^ Additionally, ion methods like LEIS can provide insights into the composition and structure of the surface layers.^16^ Scanning Probe Microscopy techniques, including STM and AFM, allow for atomic-scale imaging and topographical analysis.^17^ However, all these techniques have limitations, one very important being that NPs are susceptible to damage during analysis, which can alter their structure and properties.^17^ Furthermore, while these methods often focus on surface characteristics, they may not fully represent the core structure of multimetallic NPs.^17^ The presence of different phases and compositions can complicate the interpretation of the results. Also, the structural sensitivity of NPs to the environment further complicates characterization.^17^ While these techniques collectively enhance the understanding of chemical ordering in NPs, their limitations must be carefully considered to ensure accurate, atomically precise characterization.^16,17^

Alternatively, computational methods such as density functional theory (DFT) and MD coupled with Monte Carlo (MC) simulations offer a promising avenue for accurately assessing NP stability. Recent advances in finite-temperature DFT enable explicit inclusion of electronic and configurational entropy through free energy minimization, allowing accurate simulations of metallic NPs under realistic conditions.^18^ Thermal contributions can influence relative stability, promote configurational flexibility, and induce structural disorder.^19–21^ Previous large-scale theoretical studies on cobalt and bimetallic NPs used cohesive energy trends to predict stable configurations, despite recognizing that configurational entropy and finite-temperature effects can alter phase stability.^19,22^ Fully accounting for configurational entropy in large-sized NPs remains computationally challenging. Nonetheless, studies that explicitly incorporate configurational entropy have demonstrated that thermal effects can stabilize structures that are not enthalpically favored at 0 K, particularly in larger or compositionally complex systems.^19,21^ However, DFT encounters challenges, particularly with computational limitations in handling NPs with diameters larger than 2 nm.^23^ Additionally, it is impossible for DFT to efficiently screen through all possible chemical orderings.^24,25^ Loevlie et al. have shown that the number of potential chemical orderings increases significantly as the number of different metals increases in the NP.^24^ For instance, a 50-atom bimetallic NP with a 50/50 metal composition results in an explosion of possible chemical orderings to ∼10^14^. Hence, DFT on its own cannot overcome this limitation and for this purpose it is combined with other methods to efficiently explore the vast materials space. For example, the DFT-based topological energy expression method has been utilized to investigate the chemical ordering of a wide range of bimetallic NPs.^26,27^ In another study, the basin hopping method, the second-moment approximation to the tight-binding model, DFT, and MD were utilized to find the optimal chemical ordering of AuCo, varying the size and composition.^28^ Furthermore, the Pool-Lamarckian Birmingham Cluster Genetic Algorithm, developed in conjunction with DFT, has been used for the global optimization of nanoalloy clusters^29,30^ such as AuPd. Alternatively, cluster expansion is also commonly used to describe the chemical ordering in nanoalloys. Larsen et al. employed cluster expansion fitted to an effective theory potential along with mixed integer programming to determine the ground-state configuration in AgAu clusters.^31^ Another work carried out by Ekborg-Tanner and Erhart implemented cluster expansion along with DFT and MC simulations to examine the effect of hydrogen coverage on the mixing behavior in AuPd and PdCu systems.^32^ The combination of MC and MD simulations provides a comprehensive understanding of the effect of temperature on the chemical ordering in NPs.^33^ Wang and coworkers^34^ investigated the thermal stability of PtNi for dry reforming methane, revealing an inverse relationship between Pt segregation and temperature due to entropic effects, irrespective of shape. Ter-Oganessian et al. found that core–shell structures are more favorable at concentrations of AuPd_50_ and AuPd_75_ by employing ReaxFF in parallel with MD and MC simulations.^33^ Another study employed a DFT-trained neural network and MC to understand the segregation behavior in AuPd(111) as a function of composition and temperature.^35^ Despite these great advancements, modeling complex NP systems remains computationally expensive, as most of the current computational methods rely heavily on DFT due to its accuracy. Therefore, alternative approaches are needed to accurately and rapidly screen how NP morphology and composition affect chemical ordering.

To address the computational cost of accurately predicting the stability in NPs, Yan et al. introduced the bond-centric model (BCM).^9^ This model measures stability in terms of cohesive energy (CE), across NPs of any size, shape, and metal composition.^9^ Building on this, Dean et al. developed a genetic algorithm (GA)^36^ coupled explicitly with the BCM,^9^ facilitating efficient navigation of vast configurational space to identify thermodynamically stable chemical orderings of NPs based on their CE. Advancements like this enable rapid screening of a large search space for potential chemical orderings of multimetallic NPs. The BCM includes the bulk CE of metals (CE_bulk_), structural information represented by the coordination number of each metal atom, as well as electronic information such as the gamma value (eq 1). While all other parameters are tabulated, the gamma value can be computed by using two methods. Initially, it was calculated using primarily experimental bond dissociation energy data of metal dimers, called the “dimer method”. In the absence of experimental data, DFT could be utilized to calculate dissociation energies.^9^ However, recent efforts by Loevlie and coworkers aimed to enhance the representation of diverse coordination environments in the weight factor (gamma values) and strain effects, which were missing in the initial BCM formulation, and the method was called the “NP method”.^24^ The NP method was tested only on combinations of Au, Pt, and Pd metals on the final chemical ordering of NPs. As a result, a critical evaluation between the NP and dimer (DM) methods on the final chemical ordering predictions of NPs, as a function of metal composition and expansion on several metal combinations, is currently missing to address the accuracy of the two methods in predicting nanoscale chemical ordering.

In this work, we undertake a comparative analysis on the DM and NP methods in predicting the stability of 15 bimetallic combinations of 2869-atom NPs with a cuboctahedral shape, benchmarked against experimental and computational results. These NPs are composed of d^8^ (Pd, Pt, Ni) and d^9^ (Au, Ag, Cu) metals with systematically varying compositions. Furthermore, we extended our work to include 6 trimetallic NPs with a tremendously large materials space, keeping the size and shape constant, to evaluate the feasibility of this method in capturing overall stability and demonstrate the generalization of the BCM and GA. The study aims to critically assess and validate the predictive capabilities of the BCM coupled with the GA and to facilitate an in-depth understanding of multimetallic NP stability of any size, shape, and composition. In both bimetallic and trimetallic NPs, we systematically varied the NP composition and explored the detailed metal distribution (i.e., chemical ordering) in the core, subsurface, and surface of the NP. The insights gained from this work are intended to enhance predictions of thermodynamic stability in multimetallic NPs, to aid in the design of functional nanomaterials for a wide range of applications, and assist experimentalists in multimetallic NP characterization.

## Methodology

The chemical ordering of 2869-atom bimetallic cuboctahedron NPs composed of d^8^ and d^9^ metals of different compositions, varying by increments of 0.1 per metal (135 NP systems per gamma value method), was optimized using the GA^36^ coupled with the BCM.^9^ In this work, we focus on the cuboctahedral shape as it offers a symmetric structure with well-defined (111) and (100) facets. While the truncated octahedron is typically more stable at ∼5 nm,^37,38^ the cuboctahedron has been widely used in previous studies of surface segregation in FCC NPs^39^ and it provides a consistent geometric framework for comparing ordering trends across different bimetallic NPs. The BCM measures the stability of NPs of any size, shape, and metal composition in terms of CE_BCM_,^9^ as shown in eq 1:

1 $${\mathrm{CE}}_{\mathrm{BCM}}=\frac{\overset{m}{\underset{1}{\sum }}\gamma_{i}\times \frac{{\mathrm{CE}}_{\mathrm{bulk},i}}{{\mathrm{CN}}_{i}}\sqrt{\frac{{\mathrm{CN}}_{i}}{{\mathrm{CB}}_{i}}}+\gamma_{j}\times \frac{{\mathrm{CE}}_{\mathrm{bulk},j}}{{\mathrm{CN}}_{j}}\sqrt{\frac{{\mathrm{CN}}_{j}}{{\mathrm{CB}}_{j}}}}{n}$$

Where γ_*i*_ and γ_*j*_ (gamma values) are the weight factors for atoms *i* and *j*, respectively, CN_*i*_ and CN_*j*_ are the coordination numbers of atoms *i* and *j*, respectively, CB_*i*/*j*_ is the bulk CN of atom *i*/*j* (this work focuses on fcc metals; hence it is 12), CE_bulk,*i*/*j*_ is the bulk cohesive energy of atom *i*/*j*, and *n* is the total number of atoms in the NP, while *m* is the total number of bonds within the NP.

In the BCM, the gamma values were computed using DFT. Additionally, for the DM and NP gamma value calculations, the DM bond dissociation energy and CE of 147-atom NPs of d^8^ (Ni, Pd, and Pt) and d^9^ (Ag, Au, and Cu) metals, respectively, were calculated using CP2K,^40^ as shown below:

### DM Method

2 $${\mathrm{BDE}}_{i_{2}}\times \gamma_{i-j}+{\mathrm{BDE}}_{j_{2}}\times \gamma_{j-i}=2{\mathrm{BDE}}_{\mathrm{ij}}$$

3 $$\gamma_{i-j}+\gamma_{j-i}=2$$

Where ${\mathrm{BDE}}_{i_{2}}$ and ${\mathrm{BDE}}_{j_{2}}$ are the molecular bond dissociation energies for homometallic bonds, respectively. While ${\mathrm{BDE}}_{\mathrm{ij}}$ is the bond dissociation energy for heterometallic bonds.

### NP Method

Depending on the number of atoms (*n*) in the NP, there are two equations used to compute the gamma values:

(i) For an NP consisting of an even number of atoms

4 $${\mathrm{CE}}_{i_{k}j_{l}}={\mathrm{CE}}_{\mathrm{BCM}}{\left(\gamma_{i}\;{\mathrm{and}\;\gamma }_{j}\right)}_{i_{k}j_{l}}$$

where $k=l=\frac{n}{2}$

(ii) For an NP consisting of an odd number of atoms

5 $${\mathrm{CE}}_{i_{k}j_{l}}+{\mathrm{CE}}_{i_{l}j_{k}}={\mathrm{CE}}_{\mathrm{BCM}}{\left(\gamma_{i}\;{\mathrm{and}\;\gamma }_{j}\right)}_{i_{k}j_{l}}+{\mathrm{CE}}_{\mathrm{BCM}}{\left(\gamma_{i}\;{\mathrm{and}\;\gamma }_{j}\right)}_{i_{l}j_{k}}$$

where $k=\frac{n+1}{2}\;\mathrm{and}\;l=\frac{n-1}{2}$

Eqs 4 and 5 are basically formulations to calculate the gamma values of well-mixed bimetallic NPs (that maximize the number of heterometallic bonds) without overcounting contributions from one metal over the other. Eq 3 is then used with either eqs 4 or 5) to compute the gamma values. We acknowledge that we obtained all the gamma values for the NP method, except for Ni systems, from previous work.^24^ Also, refer to Tables S1–S3 for the values of the CE_bulk_ and gamma values used in this work. Detailed information regarding the gamma value methods, as well as the fractional coordination number, is reported in previous works.^9,24^

The fractional CNs and the radii of the monometallic 147-atom NPs were optimized to capture the interatomic strain effects using DFT, and the CN_i_ is altered to incorporate the radius of the metals (*R*_*i*_) in the 147-atom monometallic NP, as shown in eqs 6 and 7:

6 $$CN_{\mathrm{frac},i}=CN_{i}\times \frac{R_{i}}{R_{\mathrm{avg}}}$$

7 $$R_{\mathrm{avg}}=\frac{\overset{n}{\underset{i}{\sum }}R_{i}}{n}$$

Where $R_{\mathrm{avg}}$ is the average radius of atoms within the NP, and *n* is the total number of atoms in the NP.

After computing the optimized chemical ordering, there were discrepancies in the core-to-shell metal distribution in 6 bimetallic NPs (NiAg, NiAu, NiCu, NiPd, AgAu, and PtCu) between the NP and DM-gamma calculated methods (cause for critical evaluation in our study). Thus, we optimized the chemical ordering of these 6 bimetallic NPs, using a smaller NP size of 561 atoms (computationally accessible with DFT), and the GA coupled with the BCM based on the DM and NP calculated gamma values. After obtaining the optimized NPs through GA-BCM, we performed geometry optimization calculations with DFT to compute their CEs. In this way, we assessed the accuracy of the DM and NP methods for calculating chemical ordering by comparing them with DFT. We note that we chose the 561-atom NP as the GA-BCM indicates that the 561-atom NPs exhibited similar chemical ordering to that of the 2869-atom NPs (as shown in Figure S1). We then extended our work to trimetallic NPs (AuPdPt, AgAuPd, AgPdPt, AuAgPd, AuCuPd, and AgCuPd) of the same size and shape as the bimetallic cases by varying the composition with increments of 0.1. All DFT calculations were performed with the exchange correlation PBE functional in conjunction with Grimme’s D3 dispersion correction method.^41^ PBE is one of the most accurate functionals for describing bulk and surface properties of transition metals.^42^ A computational box size of 34 × 34 × 34 Å was used for all calculations. The DZVP (double-ζ valence polarized) basis set was used with the Goedecker, Teter, and Hutter (GTH) pseudopotentials at a 500 Ry cutoff.^43^ Spin polarization was also accounted for in the calculations. Self-consistent field cycles were performed with a convergence criterion of 10^–7^ Hartree. Geometry relaxations were performed using the Broyden–Fletcher–Goldfarb–Shanno minimization algorithm until the forces converged to 4.5 × 10^–4^ Hartree bohr^–1^. All initial NP structures were generated using the Atomic Simulation Environment Python package.^44^

## Results and Discussion

We applied BCM and GA to determine the most thermodynamically stable chemical ordering of the NPs. We first investigated the accuracy of the DM and NP methods in predicting chemical ordering in 15 bimetallic NP combinations. We started with Janus and random initial arrangements for each of those bimetallic NPs and optimized the structures with the GA and BCM to find the chemical ordering with the lowest CE (i.e., the most stable), as shown in Tables S4–S18. Among the 15 bimetallic combinations analyzed, the two methods showed notable agreement in predicting the most stable chemical ordering for 9 metal combinations, while 6 combinations showed discrepancies, as shown in Table 1. For the metal combinations that showed discrepancies in the chemical ordering between the NP and DM methods, we took the structure from one method and used it as an input to the other, and the GA-optimized structures consistently showed the same discrepancies (i.e., the NP method would predict a different chemical ordering from the DM method even if we used the structure of the DM method as a starting point in the GA). Additionally, we observed similarities in the segregation behavior of certain metals, which can be attributed to the intrinsic properties of the metals involved, such as the CE_bulk_. For example, Au and Ag have relatively similar properties, which, in turn, can result in similar segregation tendencies. From Table 1, AuPd and AgPd have shown that the surface of the two NPs will be rich in Au and Ag using both methods, respectively. This observation can be noticed in the rest of the bimetallic NPs with Au or Ag as one of the metals. Thus, we focus on 3 cases (out of 9) where there is no discrepancy between the methods and 3 cases (out of 6) where there is a discrepancy between the methods. In any case, all the systems are presented in the Supporting Information file.

**Table 1:** Chemical Ordering Comparison Between the NP and DM Methods

NPs	DM Chemical Ordering	NP Chemical Ordering	Match?	CE_bulk_ **Analysis**	Experimental/Computational Obs. (DM or NP)
AuPd	Au-Rich Surface	Au-Rich Surface	Yes	Au-Rich Surface	Both^45^
AuPt	Au-Rich Surface	Au-Rich Surface	Yes	Au-Rich Surface	Both^46–48^
AuCu	Au-Rich Surface	Au-Rich Surface	Yes	Au-Rich Surface	Both^49^
AgPd	Ag-Rich Surface	Ag-Rich Surface	Yes	Ag-Rich Surface	Both^50^
AgPt	Ag-Rich Surface	Ag-Rich Surface	Yes	Ag-Rich Surface	Both^48,51^
AgCu	Ag-Rich Surface	Ag-Rich Surface	Yes	Ag-Rich Surface	Both^52,53^
PdPt	Pd-Rich Surface	Pd-Rich Surface	Yes	Pd-Rich Surface	Both^54,55^
PdCu	Cu-Rich Surface	Cu-Rich Surface	Yes	Cu-Rich Surface	NA
PtNi	Pt-Rich Surface	Pt-Rich Surface	Yes	Ni-Rich Surface	Both^56^
AuAg	Mixed Surface	Ag-Rich Surface	No	Ag-Rich Surface	DM^57–59^
AuNi	Au-Rich Surface	Mixed Surface	No	Au-Rich Surface	DM^60–62^
AgNi	Ag-Rich Surface	Mixed Surface	No	Ag-Rich Surface	DM^51,63^
PtCu	Cu-Rich Surface	Mixed Surface	No	Cu-Rich Surface	DM^48,64^
PdNi	Pd-Rich Surface	Mixed Surface	No	Pd-Rich Surface	DM^65–67^
CuNi	Cu-Rich Surface	Mixed Surface	No	Cu-Rich Surface	DM^68,69^

### Agreement between DM and NP Methods

#### Palladium–Platinum (PdPt)

The chemical ordering of PdPt alloys is of significant interest due to their applications in direct methanol fuel cells. Pd-rich catalysts, such as Pd_3_Pt_1_/C, have been shown to exhibit superior selective oxygen reduction reaction (ORR) activity in the presence of methanol, preventing methanol oxidation and enhancing overall efficiency.^70^ Hence, it is crucial to investigate the chemical ordering of PdPt as a function of bulk, subsurface, and surface compositions, as illustrated in Figure 1. Since both methods resulted in similar trends, we only show the results from the DM method, while the NP method can be found in Figure S2. We find that as the Pd composition increases, Pd prefers to be on the surface, while Pt prefers to stay in the bulk and subsurface. This is more apparent in the Pd_0.5_Pt_0.5_ case, as depicted in Figure 1b,c. Compared with Pt, the surface consists of 683 Pd atoms, which corresponds to ∼84% of the surface composition. Conversely, Pd accounts for approximately 39% and 31% of the bulk and subsurface, respectively. This behavior is expected due to the difference in the CE_bulk_, which is a critical feature in the BCM. Pd exhibits a more positive CE_bulk_ (−4.2 eV/atom) compared to Pt (−6.2 eV/atom), favoring its presence on the surface. This is to maintain optimal binding among the metal constituents.^55,71^ Our predictions agree with a previous experimental study, where they found that Pd is more thermodynamically stable on the Pt surface.^54^

**Figure 1:**
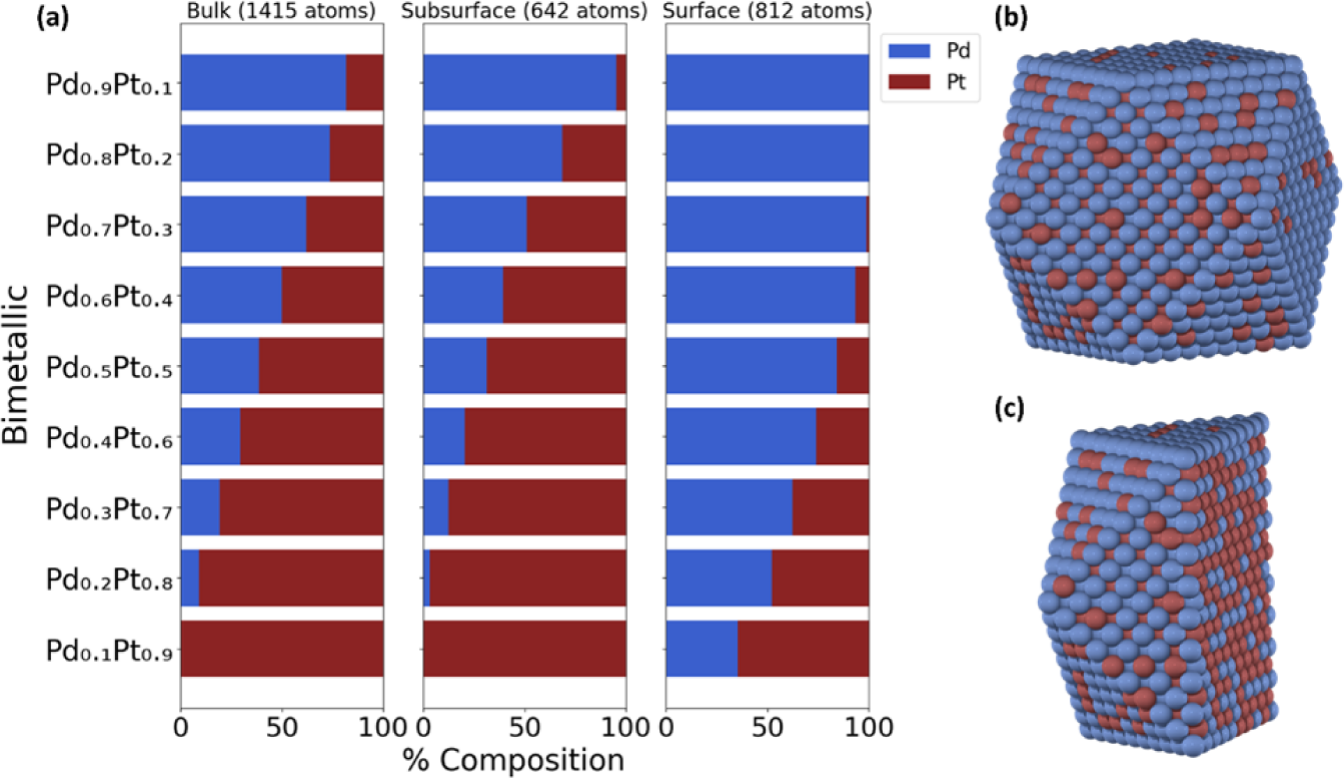
Chemical ordering of (a) a 2869-atom PdPt cuboctahedron NP as a function of bulk, subsurface, and surface composition using the DM method and a 50/50 NP composition, with its corresponding (b) surface and (c) center-cut projections.

#### Gold–Palladium (AuPd)

AuPd has gained tremendous attention due to its wide range of applications in catalysis.^72^ For instance, Pawelec et al. demonstrated that dilution of Pd with Au atoms enhances sulfur poisoning resistance by inhibiting Pd_4_S formation in the hydrodesulfurization reactions .^73^ Thus, the surface composition is critical in enhancing the catalytic properties in many reactions.^6^ We find that Au is more thermodynamically stable on the surface compared to Pd, regardless of the composition, as illustrated in Figures 2 and S3. For instance, with the compositions kept equal in both metals (Au_0.5_Pd_0.5_), Au makes up 96% of the NP surface, as depicted in Figure 2b,c. Similar to the previous case, we can anticipate these trends due to the difference in the CE_bulk_. Au has a weaker (less negative, since it is always exothermic) CE_bulk_ (−3.64 eV/atom) compared to Pd (−4.2 eV/atom), favoring its presence on the surface. These findings are consistent with observations in the literature, where a preferential surface segregation of Au in AuPd systems has been reported.^45^ Additionally, studies on the surface composition of AuPd using techniques such as Auger electron spectroscopy have revealed significant Au enrichment on the surface.^74^

**Figure 2:**
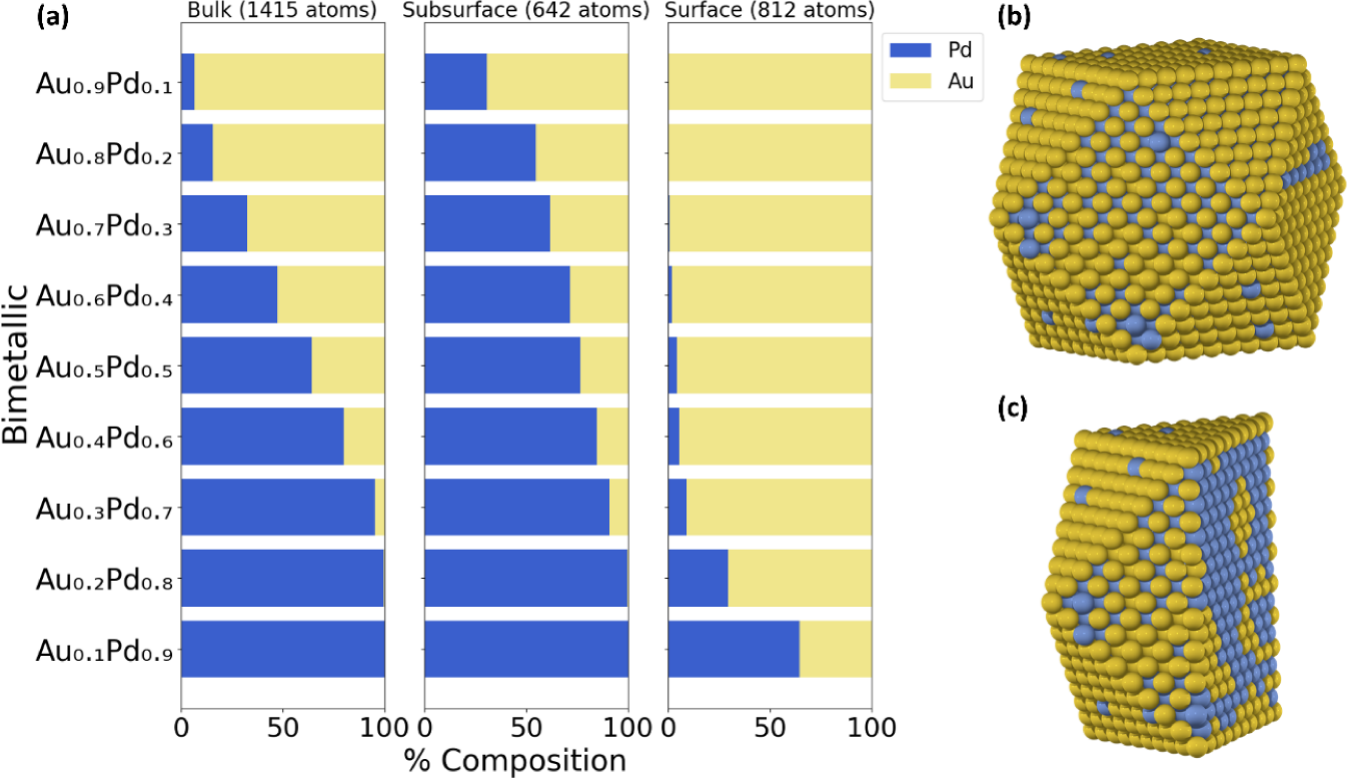
Chemical ordering of (a) a 2869-atom AuPd cuboctahedron NP as a function of bulk, subsurface, and surface composition using the DM method and a 50/50 NP composition, with its corresponding (b) surface and (c) center-cut projections.

#### Silver–Copper (AgCu)

AgCu NPs have been shown to catalyze various reactions, such as ammonia oxidation^75^ to nitrogen and the ORR.^76^ The performance of the catalyst can be tuned via its composition to enhance its catalytic performance. For instance, Zhang et al. demonstrated that high concentrations of Cu on the surface enhanced the selectivity of N_2_ by 15%, whereas dilute Cu on Ag exhibited enhanced adsorption of O_2_.^76^ Therefore, accurately predicting the chemical ordering in AgCu is important for optimizing these catalytic properties. Both (DM and NP) methods predict that Ag dominates the surface compared to Cu, as illustrated in Figures 3 and S4. Specifically, even with an equal composition of Ag and Cu (Ag_0.5_Cu_0.5_), nearly 98% of the surface is filled with Ag. This observation can be attributed to the difference in CE_bulk_, as Ag (−2.96 eV/atom) possesses a weaker CE_bulk_ compared with Cu (−3.95 eV/atom). Furthermore, it is evident that regardless of the composition, Ag tends to occupy the surface. This was also confirmed in the literature, where Ag is present on the shell of the AgCu NPs through a combination of micro-Raman spectroscopy and transmission electron microscopy analysis, which allowed them to distinguish between Cu and Ag^52^.

**Figure 3:**
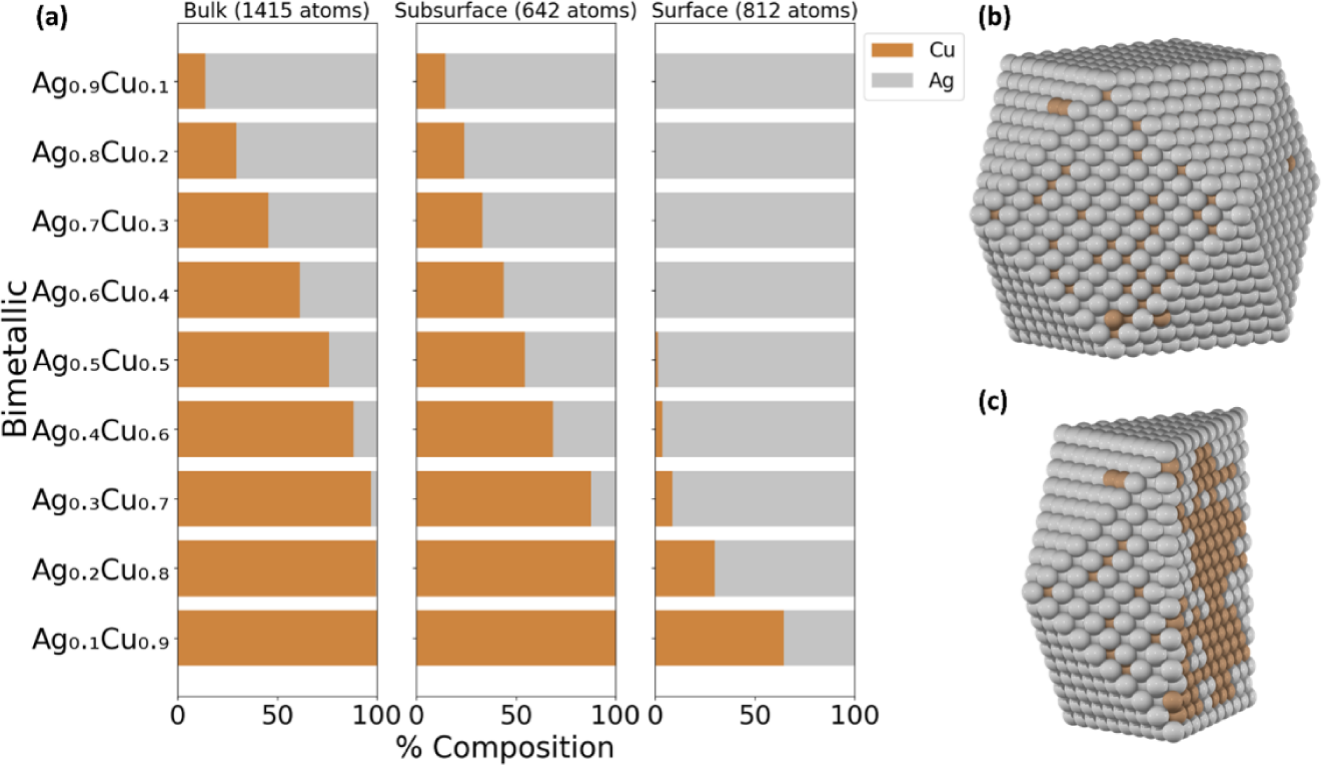
Chemical ordering of (a) a 2869-atom AgCu cuboctahedron NP as a function of bulk, subsurface, and surface composition using the DM method and a 50/50 NP composition, with its corresponding (b) surface and (c) center-cut projections.

The remaining DM and NP method results can be found in Figures S5–S10). Next, we examine the 6 cases that showed different results when comparing the optimized chemical ordering between both methods. As mentioned earlier, we will discuss 3 out of the 6 cases: AgNi, AuAg, and AuNi NPs, while the remaining 3 cases can be found in Figures S11–S13.

### Discrepancy between DM and NP Methods

#### Silver–Nickel (AgNi)

AgNi NP are commonly used in the field of electronics, particularly as conductive paste, owing to the favorable surface characteristics of Ag, such as high thermal and electrical conductivity,^77,78^ coupled with the magnetic properties of Ni. The presence of Ag on the surface is critical for preventing the oxidation of Ni, highlighting the importance of understanding the atomic arrangement in these NPs. According to predictions from the NP method, a relatively mixed surface of Ag and Ni is observed, richer in Ag, whereas the DM method indicates a surface predominantly rich in Ag, as depicted in Figures 4a,b. This trend can be seen in the Ag_0.5_Ni_0.5_ NP, in Figure 4c,d. More specifically, the DM method, as illustrated in Figure 4a, shows that the bulk consists of ∼21% Ag, the subsurface consists of ∼57% Ag, and the surface consists of ∼95% Ag. Conversely, the NP method predicts that the bulk consists of ∼44% Ag, the subsurface of ∼45% Ag, and the surface of ∼65%, as depicted in Figure 1b. Even at low concentrations of Ag (e.g., Ag_0.1_Ni_0.9_), the DM method consistently indicates a preference for Ag to reside entirely on the surface, unlike the NP method, which predicts some population in the subsurface. Based on the CE_bulk_ analysis, it is expected that Ag (−2.96 eV/atom) will prefer to be on the surface compared to Ni (−5.11 eV/atom), as is pronounced in the DM method. The presence of Ag on the NP surface is consistent with experimental observations indicating that Ag atoms are predominantly located on the shell of the AgNi NPs.^63^ Overall, the experimental observations support the fact that Ag is on the surface, which is profoundly predicted by the DM method.

**Figure 4:**
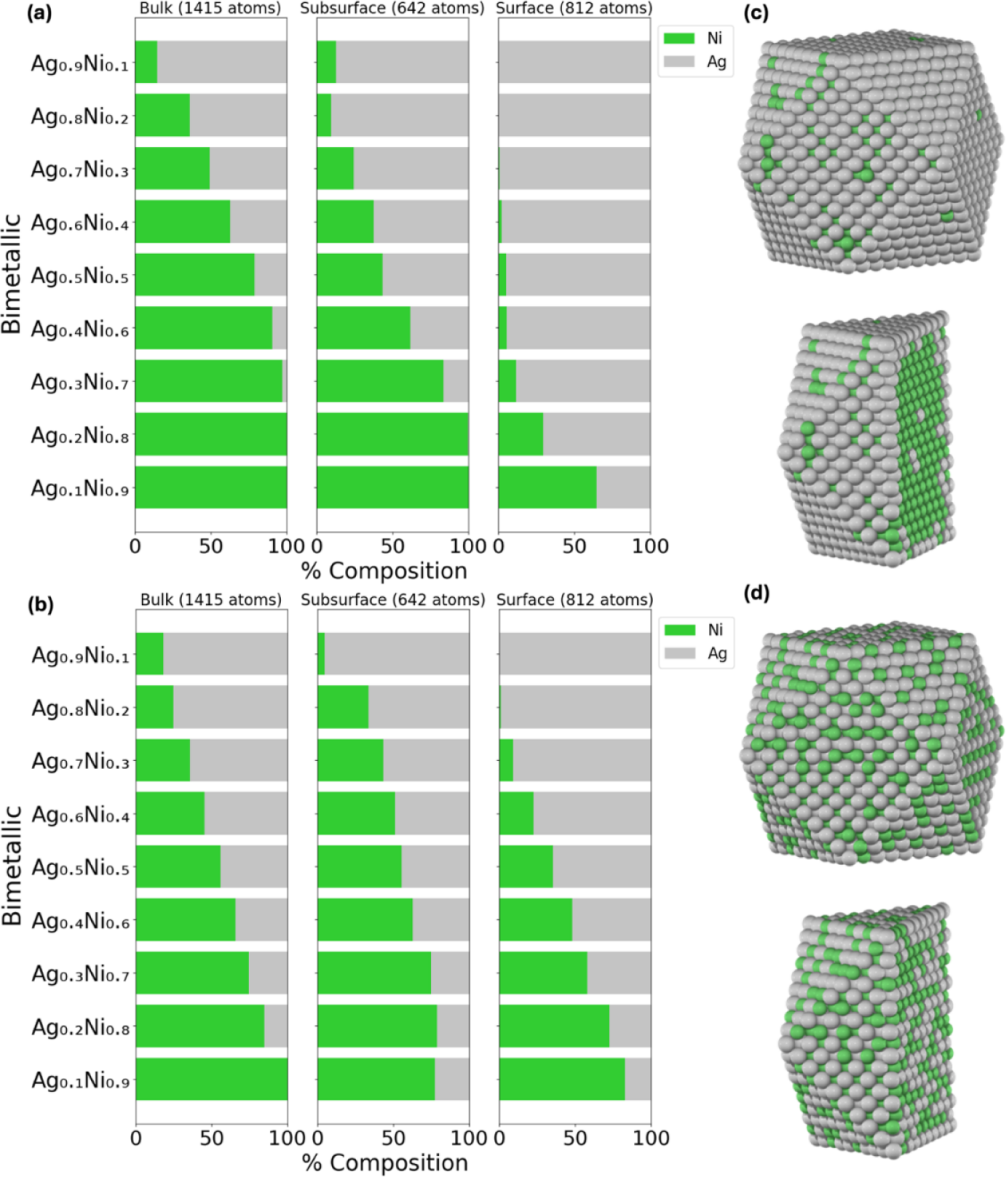
Chemical ordering of a 2869-atom AgNi cuboctahedron NP as a function of bulk, subsurface, and surface composition using the (a) DM and (b) NP methods and a 50/50 NP composition, with its corresponding surface and centered-cut projections obtained with (c) DM and (d) NP methods.

#### Gold–Silver (AuAg)

AuAg NP find widespread applications, including cancer therapy^79^ and catalysis,^80^ due to their plasmonic^81^ and catalytic properties.^82^ A recent study has shown that the metal core and surface composition are crucial in determining the peroxidase nanozyme activity,^83^ hence, we need to examine the effect of composition on the chemical ordering in AuAg. According to the DM method results, there is a mixed surface composition, whereas the NP method predicts that Ag predominantly occupies the surface, as illustrated in Figure 5a,b, respectively. The observations from the DM method can be explained by the similarities in the lattice constants between Au (*a*_0_ = 4.117 Å) and Ag (*a*_0_ = 4.091 Å),^84^ achieving complete miscibility at any composition in the bulk and nanoscale.^57^ Furthermore, experimental studies have confirmed the formation of AuAg alloys, as observed with the DM method.^57–59^

**Figure 5:**
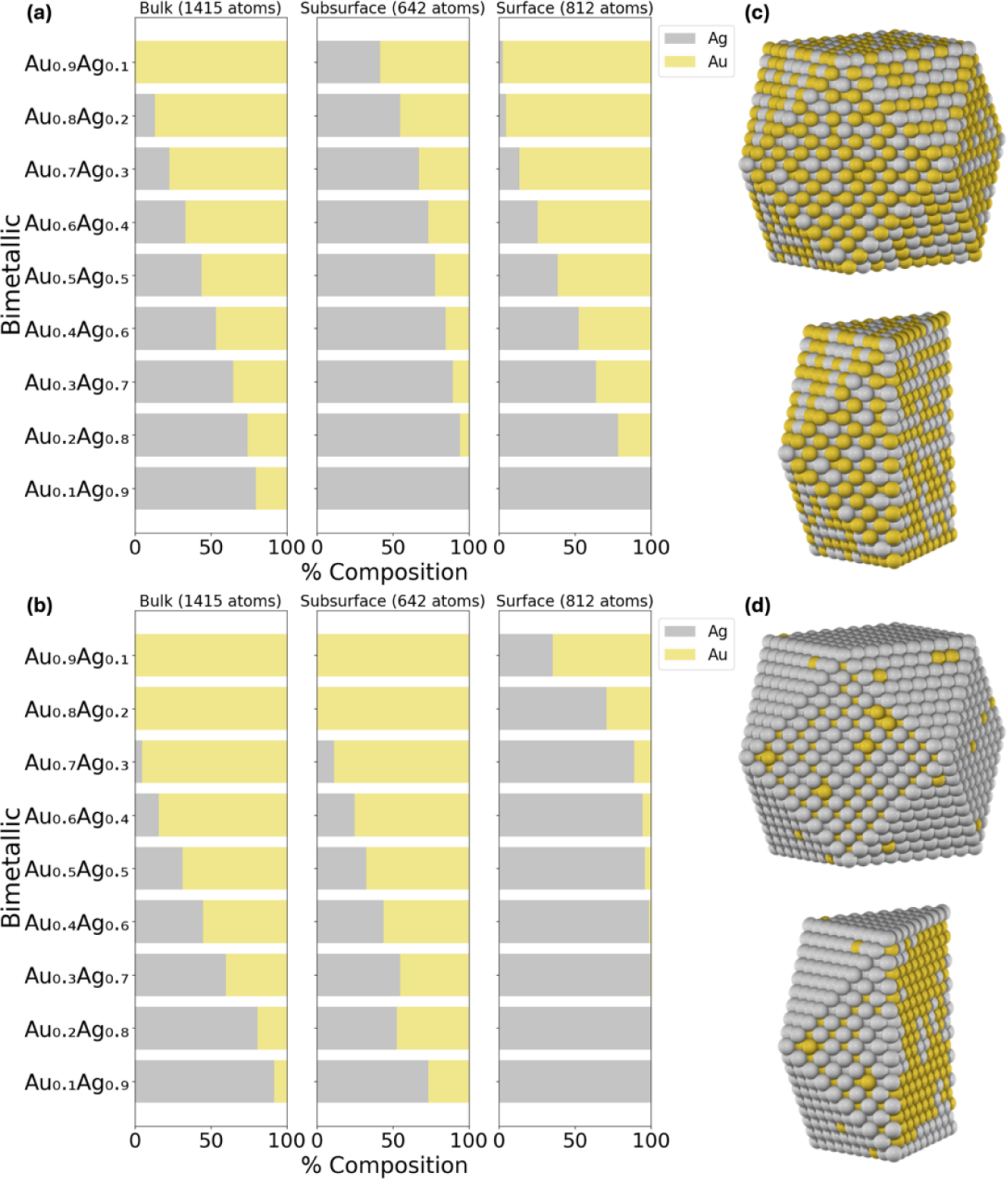
Chemical ordering of a 2869-atom AuAg cuboctahedron NP as a function of bulk, subsurface, and surface composition using the (a) DM and (b) NP methods and a 50/50 NP composition, with its corresponding surface and centered-cut projections obtained with (c) DM and (d) NP methods.

#### Gold–Nickel (AuNi)

The AuNi NP serves as a highly effective catalyst for the selective hydrogenation of butadiene, showcasing significant advantages over monometallic Au and Ni NP catalysts.^85^ When comparing the NP and DM methods, we find a clear discrepancy between the two methods, especially in the surface composition, as depicted in Figure 6a,b. This difference is particularly pronounced in the Au_0.5_Ni_0.5_ NP case, as shown in Figure 6c,d. The DM method indicates a Au-rich surface (approximately 98%), whereas the NP method predicts a mixed surface composition. The analysis based on the CE_bulk_ aligns with the trends from the DM method, indicating that Au (−3.64 eV/atom) is expected to dominate the surface as opposed to Ni (−5.11 eV/atom). This trend is further supported by experimental XPS results on Au_0.5_Ni_0.2_, where Au was found to preferentially occupy the surface.^61^

**Figure 6:**
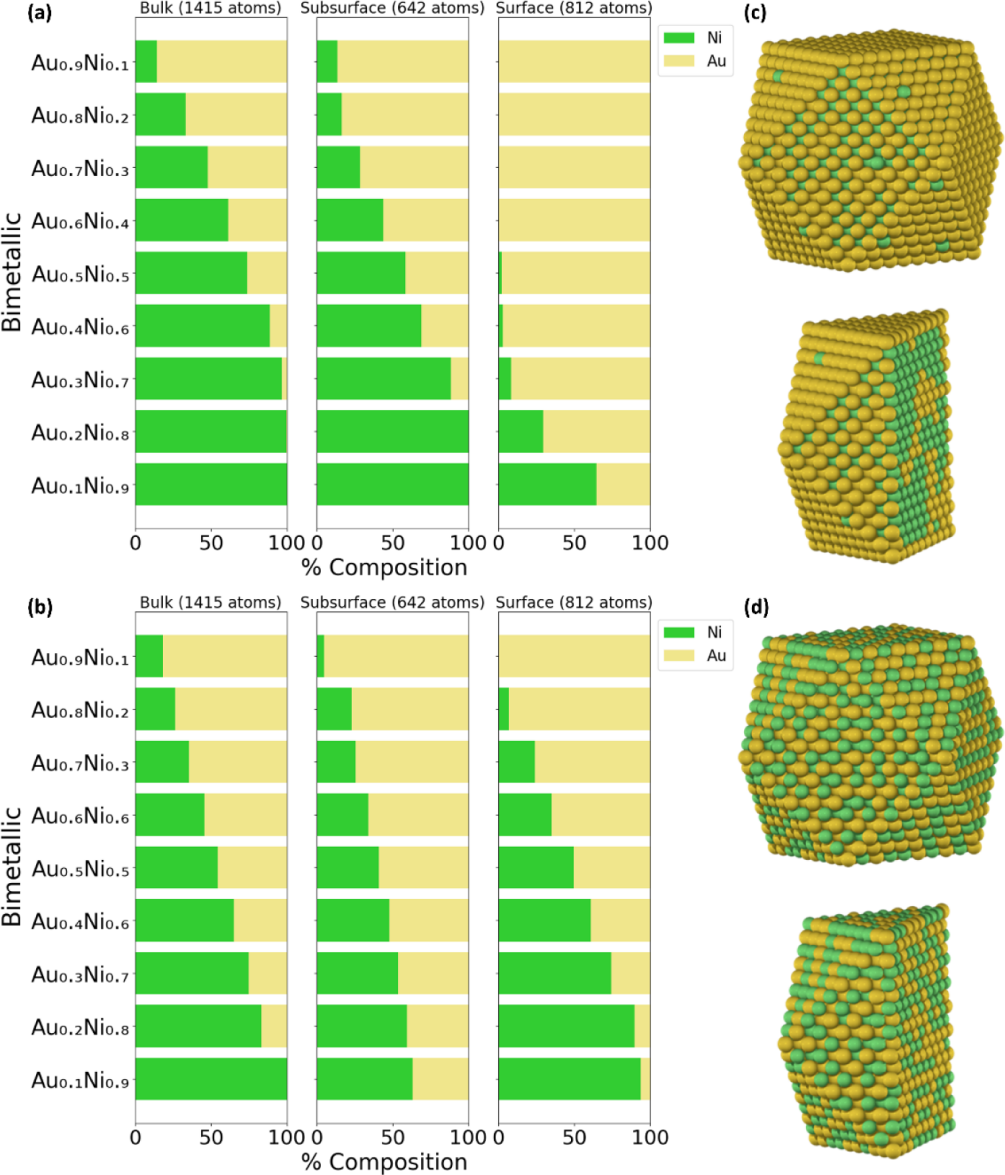
Chemical ordering of a 2869-atom AuNi cuboctahedron NP as a function of bulk, subsurface, and surface composition using the (a) DM and (b) NP methods and a 50/50 NP composition, with its corresponding surface and centered-cut projections obtained with (c) DM and (d) NP methods.

The differences we have seen so far between these two methods lie in their approach to calculate gamma values in BCM. While the NP method considers the diverse coordination environments within NPs and incorporates strain effects by introducing a modified coordination number (fractional CN), the DM method, with its simplicity in considering DM bond dissociation energies, has proven to be a superior method compared to the NP method. In addition to the computational and experimental observations, we further confirmed our results through DFT by calculating the CE of 561 atoms of 50/50 composition for the optimized GA and BCM NP from both methods. All DFT-optimized structures are provided in Figure S14. Overall, we show that even with DFT, the DM method accurately captured the most thermodynamically stable chemical ordering, as evident from Table S20.

### Extension to Trimetallic NP

Given the accuracy of the DM-calculated gamma values in the BCM coupled with the GA, we expanded our study to 6 trimetallic combinations. In this section, we will focus on discussing 2 of these 6 combinations: AuPdPt and AgAuPd. The rest of the combinations can be found in Figures S15–S18. As with the previous cases, we investigated the effect of composition on the chemical ordering in these trimetallic NPs.

#### Gold–Palladium–Platinum (AuPdPt)

The AuPdPt electrocatalyst on tungsten carbide (WC) outperforms platinum on carbon (Pt/C) in methanol oxidation, offering superior activity and stability. It shows better performance at higher temperatures and a lower activation energy. This makes AuPdPt@WC a promising alternative for direct methanol fuel cells.^86^ The visual representation of the chemical ordering for the AuPdPt NP is illustrated in Figure 7a. Interestingly, we can make predictions on the chemical ordering of trimetallic systems by leveraging the chemical ordering observed in the corresponding bimetallic cases. For instance, in the AuPd and AuPt NPs, we observe that Au prefers to reside on the surface (depicted in Figures 2 and S8a). Additionally, at lower percentages of Au, Pd, and Pt dominate the sublayer, exhibiting behavior similar to that of Pt and Pd in the bimetallic PdPt case (as shown in Figure 1). Like the bimetallic cases, we can rationalize the results based on the CE_bulk_ analysis. Based on the order of the CE_bulk_ with |CE _bulk‑Au_| < |CE _bulk‑Pd_| < |CE _bulk‑Pt_|, we infer that Au will predominantly exist on the surface, followed by Pd and Pt. This trend is more clearly observed in the Au_0.4_Pd_0.3_Pt_0.3_ (shown in Figure 7b,c) and Au_0.2_Pd_0.4_Pt_0.4_ cases, where more Au will be on the surface compared to Pd and Pt, and more Pd will be on the subsurface compared to Pt, while the core is predominantly composed of Pt. These observations align with computational studies using the Gupta potential and MC simulations.^87,88^ Additionally, we can extend our predictions to the AgPdPt. Since Ag and Au are similar, especially in their lattice constants,^57^ we observe that Ag is more stable on the NP surface, while Pd and Pt are more likely to be found in the sublayer and bulk, as illustrated in Figure S15.

**Figure 7:**
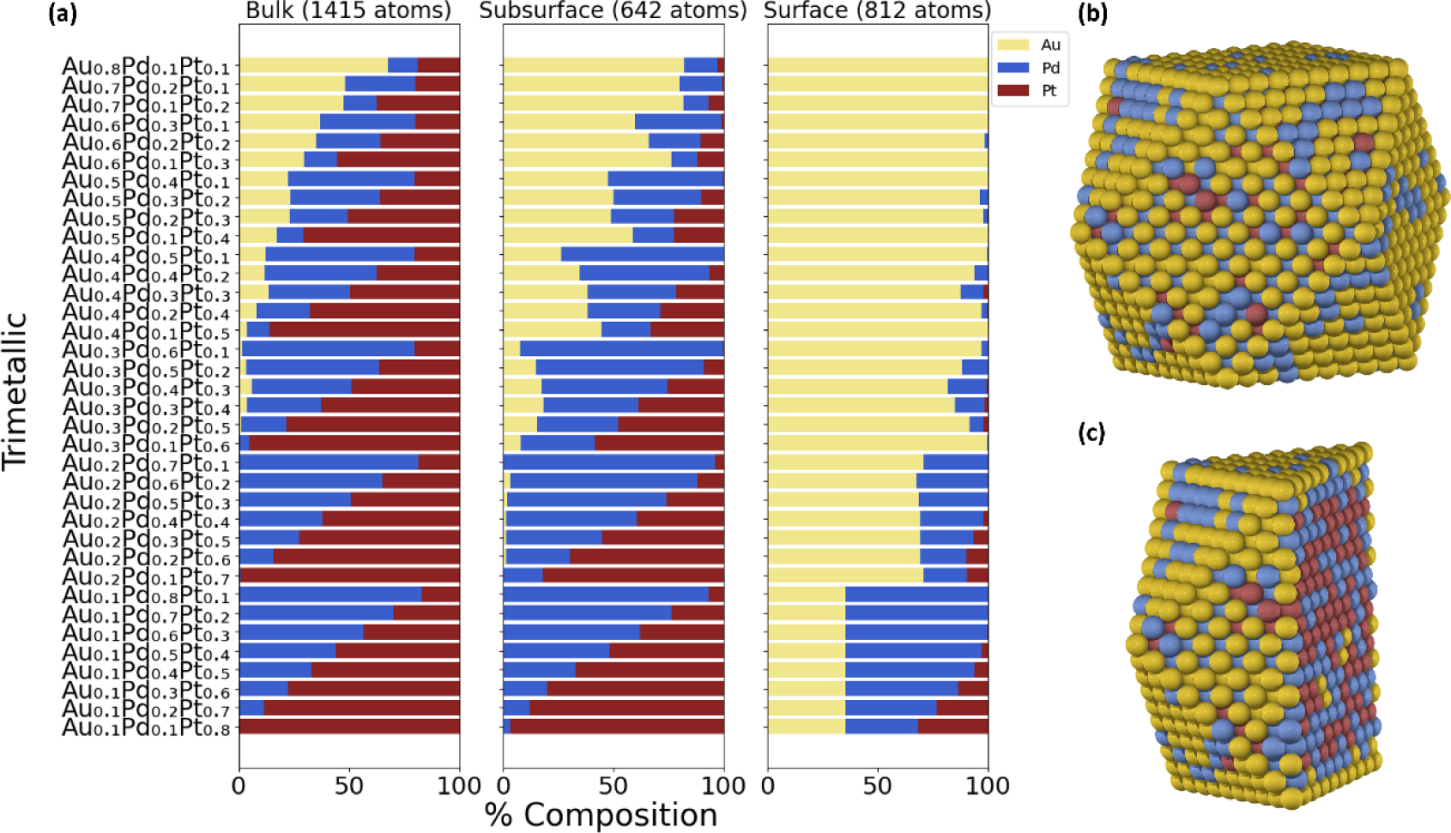
Chemical ordering of (a) a 2869-atom AuPdPt cuboctahedron NP as a function of bulk, subsurface, and surface composition using the DM method and the NP with Au_0.4_Pd_0.3_Pt_0.3_ composition, with its corresponding (b) surface and (c) center-cut projections.

#### Gold–Silver–Platinum (AuAgPt)

AgAuPt NPs exhibit superior catalytic activity for glucose oxidation compared to both their monometallic and bimetallic counterparts.^89^ Following the same approach as in the AuPdPt NPs, we find that the chemical ordering in AuAgPt NPs (Figure 8) can also be estimated using the predictions from the bimetallic combinations (shown in Figures 5a, S6a, and S8a). In this system, Ag and Au are expected to segregate to the surface due to the similarities between Ag and Au^57^, while Pt resides in the subsurface and bulk layers, as observed in Figure 8a. This order correlates with the increasing CE_bulk_, with |CE _bulk‑Ag_| < |CE_bulk‑Au_| < |CE_bulk‑Pt_|, demonstrating how CE_bulk_ captures stability in multimetallic NPs through the BCM. This is clearly depicted in the case of Au_0.3_Ag_0.3_Pt_0.4_ NP (Figure 8b,c). However, at high concentrations of Pt (∼0.8), small amounts of Pt may appear on the surface as the bulk and sublayer are already almost filled with Pt. A study conducted by Pacheco-Contreras et al. utilized Gupta potential and DFT calculations to optimize the trimetallic AgAuPt clusters. In these clusters, there is a noticeable observation for Ag and Au atoms to be on the surface, while Pt atoms tend to fill the core.^90^ Similarly, the AuAgPd system exhibits a pattern similar to that of AuAgPt, as demonstrated in Figure S16.

**Figure 8:**
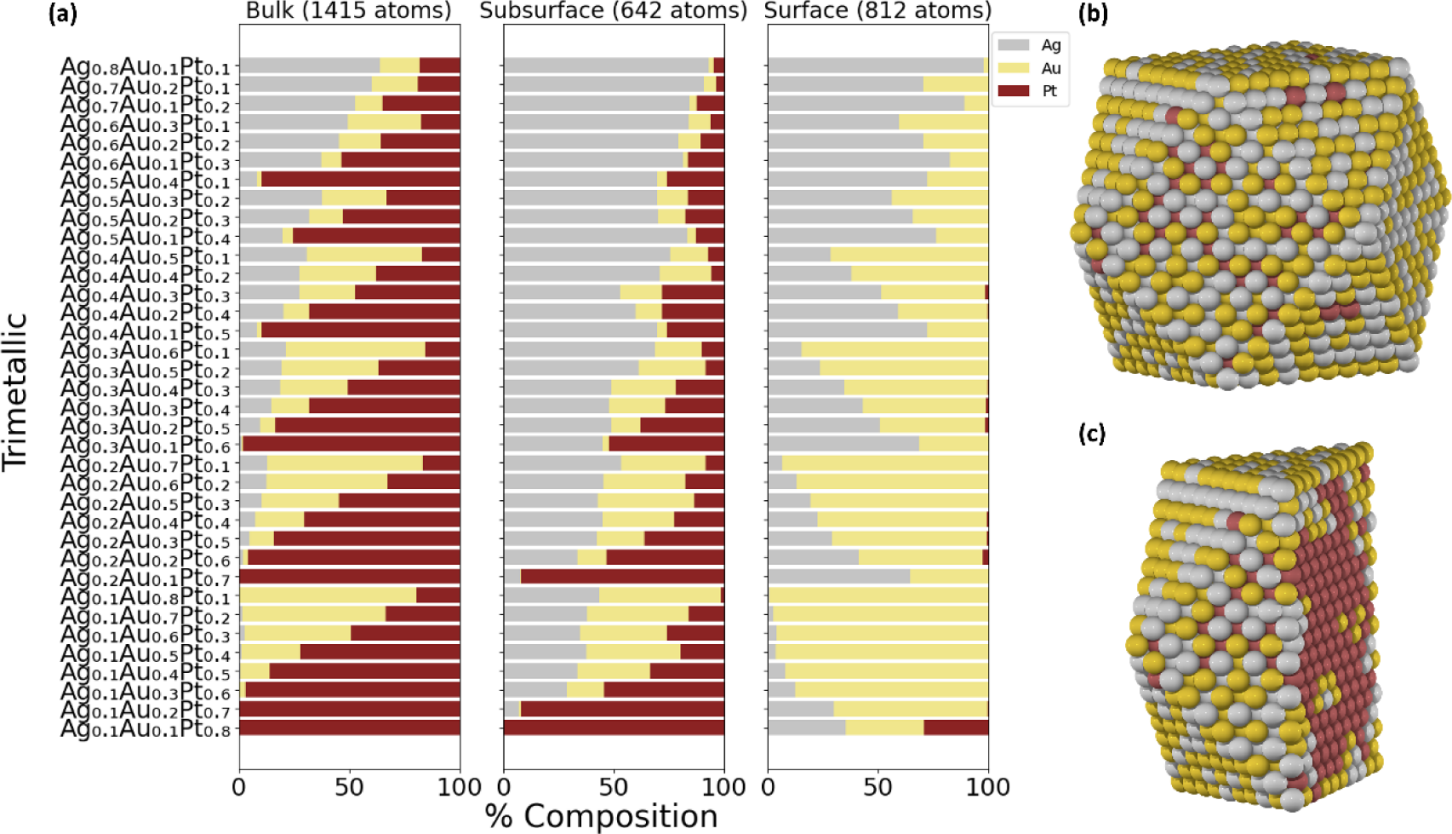
Chemical ordering of (a) a 2869-atom AuAgPt cuboctahedron NP as a function of bulk, subsurface, and surface composition using the DM method and the NP with Au_0.3_Ag_0.3_Pt_0.4_ composition, with its corresponding (b) surface and (c) center-cut projections.

As a final note, all of our predictions have been compared against experimental data. We acknowledge that in experimental NP synthesis, achieving thermodynamic equilibrium can be challenging due to kinetic constraints that often prevent NPs from reaching their most stable configuration. To address this, we made a concerted effort to avoid experimental data that contain a chemical environment that could influence chemical ordering within the NPs. We specifically excluded analyses of experiments that used stabilizing or reducing agents or ligands, and we avoided relying on experiments that involved sequential processes. This was done to ensure that our comparisons were made against NPs that most closely represent thermodynamic equilibrium under the given experimental conditions.

We would also like to note that in metal combinations with large lattice mismatch, strain effects may lead to significant distortions, which, in turn, can lead to structural transitions.^91,92^ Our approach applies the rigid-lattice framework and, therefore, does not account for geometry relaxation. Nevertheless, our approach enables systematic and rapid comparison of thermodynamic stability in terms of chemical ordering changes and captures the surface compositions for a wide range of bimetallic and trimetallic NPs, even with moderate lattice mismatch. In cases where there is large lattice mismatch, one can use the fractional CN (accounting for metal distances in the gamma values calculations) and apply two-step NP optimization strategies, where the chemical ordering is first optimized (at a given NP shape) and then the NP shape, similar to approaches developed for bimetallic clusters.^93^

## Conclusion

In this work, we employed BCM and GA to predict the stability of bimetallic and trimetallic NPs across a range of metal compositions. Initially, we evaluated the accuracy of the NP and DM-gamma calculated methods within the BCM. We focused on comparing the traditional DM method to the newly developed NP method. This involved optimizing the chemical ordering of 2869-atom cuboctahedron NPs for 15 bimetallic combinations using the GA and the BCM. Our results revealed a notable agreement between the two methods in 9 out of the 15 combinations, while the remaining 6 combinations showed discrepancies. Despite the ability of the NP method to account for a variety of coordination environments and strain effects, the DM method proved to be more accurate and computationally efficient for a wide range of metal combinations when benchmarked against experimental and computational results. Loevlie et al.^24^ utilized the PBE functional with D3 correction to develop the NP method for calculating gamma values but compared it against the DM predictions, where the gamma values were computed from experimental bond dissociation energies. Our results suggest that the DM method should be used with PBE-D3, indicating accurate chemical ordering trends. This key difference in methodology explains the observed difference between our current and previous work^24^ and demonstrates that one should consistently rely on PBE-D3 calculations for gamma values, rather than experimental bond dissociation energies.

Following our work on bimetallics, we expanded our investigation to include 6 trimetallic NPs, keeping the size and shape constant, using the DM-calculated gamma values. The results revealed consistent and predictable patterns in the distribution of metals within the NPs based on the chemical ordering of the bimetallic NPs. Additionally, this study also highlights the critical role of CE_bulk_ values in the BCM in predicting the chemical ordering, where metals with lower CE_bulk_ tend to occupy the surface, and vice versa.

To accurately predict the chemical ordering of multimetallic NPs using the BCM coupled with the GA, we suggest the following steps/rules to users:

• The GA code and related package should be downloaded and installed from the GitHub repository.^94^

• As illustrated in this work, DM gamma values should be used in the BCM based on bond dissociation values calculated using the PBE-D3 functional. A complete list of the gamma values is available in our GitHub repository.^94^

• The CE_bulk_ values have been obtained from previously published work^84,95^ and we consistently used PBE-D3-derived CE_bulk_ values throughout our analysis, as it shows accuracy with respect to transition metals^42^ and are also consistent with the PBE-D3-derived DM gamma values.

• Since we found that the gamma value calculated using the DM method outperformed the NP method, we use the simple integer CN representation in the BCM. However, the fractional CN representation, as used in the NP method, can still be used in cases where there is a large size difference between the metals and can be compared against the DM method in terms of chemical ordering predictions.

• The maximum number of generations in the GA can be set to 10,000, as we found that the chemical ordering results converged by this point for the system sizes we investigated.

These guidelines ensure a consistent application of our methodology for accurately predicting the stability and chemical ordering of multimetallic NPs. Overall, our study offers a computationally efficient alternative to traditional simulations, enabling the rapid and efficient screening of NP compositions and morphologies, and predicting NPs with highly stable chemical ordering that is central for various nanotechnological applications.

## Supplementary Material



## Notes

Detailed methodology for gamma value calculations; cohesive energy and gamma values for all studied metals; complete chemical ordering for 15 bimetallic and 6 trimetallic nanoparticles; additional chemical ordering figures for all nanoparticle systems; DFT-optimized structures for selected nanoparticles; and integration of machine learning with physics; comparative analysis between dimer- and nanoparticle-based gamma value methods (PDF)

## ∇

School of Chemical Engineering National Technical University of Athens (NTUA) Athens GR-15780 Greece
