# Supplementary material for: Rapid Atomic Structure Prediction of Multimetallic Nanoparticles with Physics-Based Machine Learning
Source: ACS Omega. 2025 Jul 14;10(28):30996–1008. doi: 10.1021/acsomega.5c04082 (PMC12290662; doi:10.1021/acsomega.5c04082)
Supplement: Supplementary file 1 [file ao5c04082_si_001.pdf]

Supporting Information

Rapid Atomic Structure Prediction of Multimetallic Nanoparticles with Physics-based Machine Learning

Bassel Alkhatib, Maya Salem, Klaertje Kiyora Hesselink, and Giannis Mpourmpakis[^],*

Department of Chemical and Petroleum Engineering, University of Pittsburgh, Pittsburgh, Pennsylvania 15261, United States

[^]Present Address: School of Chemical Engineering, National Technical University of Athens (NTUA), Athens, GR-15780, Greece

*Corresponding author. E-mail: gmpourmp@pitt.edu

1. CE_{bulk} and Gamma Values Tables

Table S1. CE_{bulk} values used in the BCM for different metals.

Metal	CE_{bulk} (eV/atom)
Au	-3.64
Ag	-2.96
Pd	-4.2
Pt	-6.2
Cu	-3.95
Ni	-5.11

Table S2. Gamma values calculated using the NP method.

**Au**	{Au: 1}<br>{Ag: 0.754}<br>{Pd: 2.945}<br>{Pt: 0.972}<br>{Cu: 0.890}<br>{Ni: 0.416}	**Ag**	{Au: 1.246}<br>{Ag: 1}<br>{Pd: 1.489}<br>{Pt: 0.896}<br>{Cu: 0.956}<br>{Ni: 0.613}	**Pd**	{Au: -0.945}<br>{Ag: 0.511}<br>{Pd: 1}<br>{Pt: 1.187}<br>{Cu: 0.293}<br>{Ni: 0.624}
**Pt**	{Au: 1.028}<br>{Ag: 1.104}<br>{Pd: 0.813}<br>{Pt: 1}<br>{Cu: 1.455}<br>{Ni: 2.733}	**Cu**	{Au: 1.110}<br>{Ag: 1.044}<br>{Pd: 1.707}<br>{Pt: 0.544}<br>{Cu: 1}<br>{Ni: 0.275}	**Ni**	{Au: 1.584}<br>{Ag: 1.387}<br>{Pd: 1.376}<br>{Pt: -0.733}<br>{Cu: 1.725}<br>{Ni: 1}

Table S3. Gamma values calculated using the DM method.

**Au**	{Au: 1}<br>{Ag: 1.525}<br>{Pd: 1.242}<br>{Pt: 1.301}<br>{Cu: 2.996}<br>{Ni: 1.020}	**Ag**	{Au: 0.475}<br>{Ag: 1}<br>{Pd: 1.060}<br>{Pt: 1.181}<br>{Cu: 1.039}<br>{Ni: 1.258}	**Pd**	{Au: 0.758}<br>{Ag: 0.940}<br>{Pd: 1}<br>{Pt: 0.818}<br>{Cu: 0.733}<br>{Ni: 1.261}
**Pt**	{Au: 0.699}<br>{Ag: 0.819}<br>{Pd: 1.182}<br>{Pt: 1}<br>{Cu: 1.088}<br>{Ni: 1.768}	**Cu**	{Au: -0.996}<br>{Ag: 0.961}<br>{Pd: 1.267}<br>{Pt: 0.912}<br>{Cu: 1}<br>{Ni: 1.260}	**Ni**	{Au: 0.980}<br>{Ag: 0.742}<br>{Pd: 0.739}<br>{Pt: 0.232}<br>{Cu: 0.740}<br>{Ni: 1}

(Example: The gamma value of A-B indicates how atom A contributes to the bond shared with atom B. For a bimetallic NP of AuPd, the gamma value of Au-Pd = 2.945 and the gamma value of Pd-Au = -0.945 using the NP method, reflecting asymmetric bond contributions. This asymmetry arises because Au and Pd contribute differently to the bond strength, with Au having a greater weighting factor in Au-Pd than Pd does in Pd-Au.)

2. CE starting from Janus and random NP chemical ordering

We compared the CE of the optimized chemical ordering produced from two different initial structures: (1) Janus and (2) random structures. Based on the lowest CE (bold), we use this NP in our analysis. Note that the optimized NP (bold) does not necessarily remain in the same chemical ordering as the initial structure. We used different initial starting points (Janus and random) to reach the lowest optimized chemical ordering. Both initial structures eventually will result in an "almost" similar chemical ordering for the final optimal NP.

Table S4. Optimized AgCu NP CE starting from Janus and random initial structures using the NP and DM methods.

				NP method CE (eV/atom)		DM method CE (eV/atom)	
Metal i	Composition	Metal j	Composition	Janus	Random	Janus	Random
Ag	0.1	Cu	0.9	**-3.67949**	-3.67948	-3.66607	**-3.66608**
Ag	0.2	Cu	0.8	-3.60677	**-3.6068**	**-3.58354**	-3.58339
Ag	0.3	Cu	0.7	-3.52302	**-3.52343**	-3.49142	**-3.49232**
Ag	0.4	Cu	0.6	**-3.4316**	**-3.4316**	-3.39247	**-3.3939**
Ag	0.5	Cu	0.5	**-3.33568**	-3.33556	-3.29484	**-3.29514**
Ag	0.6	Cu	0.4	-3.23732	**-3.23739**	**-3.19658**	-3.19652
Ag	0.7	Cu	0.3	**-3.1345**	-3.13448	**-3.09878**	-3.09853
Ag	0.8	Cu	0.2	**-3.02814**	-3.02812	**-3.00083**	-3.00052
Ag	0.9	Cu	0.1	-2.9184	**-2.91843**	**-2.9036**	-2.90329

Table S5. Optimized CuPd NP CE starting from Janus and random initial structures using the NP and DM methods.

				NP method CE (eV/atom)		DM method CE (eV/atom)	
Metal i	Composition	Metal j	Composition	Janus	Random	Janus	Random
Cu	0.1	Pd	0.9	-3.97896	**-3.97897**	**-3.96639**	-3.96633
Cu	0.2	Pd	0.8	-3.97182	**-3.97217**	-3.94981	**-3.94984**
Cu	0.3	Pd	0.7	**-3.94886**	-3.94777	**-3.92567**	-3.92531
Cu	0.4	Pd	0.6	**-3.92319**	-3.92264	-3.89822	**-3.89852**
Cu	0.5	Pd	0.5	-3.89529	**-3.8975**	-3.87182	**-3.87195**
Cu	0.6	Pd	0.4	**-3.87507**	-3.87388	**-3.84766**	-3.84696
Cu	0.7	Pd	0.3	-3.85183	**-3.852**	**-3.82422**	-3.82397
Cu	0.8	Pd	0.2	-3.8268	**-3.82721**	-3.80049	**-3.80052**
Cu	0.9	Pd	0.1	-3.79007	**-3.79008**	-3.77347	**-3.77348**

Table S6. Optimized PtNi NP CE starting from Janus and random initial structures using the NP and DM methods.

				NP method CE (eV/atom)		DM method CE (eV/atom)	
Metal i	Composition	Metal j	Composition	Janus	Random	Janus	Random
Pt	0.1	Ni	0.9	-5.09975	**-5.10014**	-5.03471	**-5.03473**
Pt	0.2	Ni	0.8	**-5.33208**	-5.32889	-5.21474	**-5.21563**
Pt	0.3	Ni	0.7	**-5.52552**	-5.5244	-5.37176	**-5.37197**
Pt	0.4	Ni	0.6	-5.68596	**-5.68753**	**-5.50688**	-5.50278
Pt	0.5	Ni	0.5	-5.79793	**-5.81572**	**-5.62358**	-5.61967
Pt	0.6	Ni	0.4	**-5.92944**	-5.92701	-5.72107	**-5.72413**
Pt	0.7	Ni	0.3	-5.99299	**-5.99574**	-5.80397	**-5.8053**
Pt	0.8	Ni	0.2	**-6.01344**	-6.01226	-5.85677	**-5.8578**
Pt	0.9	Ni	0.1	-5.98421	**-5.98482**	-5.88595	**-5.88623**

Table S7. Optimized AuPt NP CE starting from Janus and random initial structures using the NP and DM methods.

				NP method CE (eV/atom)		DM method CE (eV/atom)	
Metal i	Composition	Metal j	Composition	Janus	Random	Janus	Random
Au	0.1	Pt	0.9	**-5.69019**	-5.69016	-5.65544	**-5.656**
Au	0.2	Pt	0.8	**-5.48758**	-5.48627	-5.4201	**-5.42239**
Au	0.3	Pt	0.7	**-5.26091**	-5.25916	**-5.16523**	-5.16256
Au	0.4	Pt	0.6	**-5.01851**	-5.01786	**-4.89818**	-4.89245
Au	0.5	Pt	0.5	**-4.76696**	-4.76691	**-4.62381**	-4.62246
Au	0.6	Pt	0.4	-4.51377	**-4.51383**	**-4.38098**	-4.37406
Au	0.7	Pt	0.3	-4.25415	**-4.25428**	**-4.14283**	-4.14088
Au	0.8	Pt	0.2	**-3.99013**	-3.99002	-3.91703	**-3.92078**
Au	0.9	Pt	0.1	**-3.72179**	-3.72176	**-3.68636**	-3.68596

Table S8. Optimized AuPd NP CE starting from Janus and random initial structures using the NP and DM methods.

				NP method CE (eV/atom)		DM method CE (eV/atom)	
Metal i	Composition	Metal j	Composition	Janus	Random	Janus	Random
Au	0.1	Pd	0.9	**-3.9428**	-3.94161	**-3.93834**	-3.9383
Au	0.2	Pd	0.8	**-3.89536**	-3.89446	-3.89189	**-3.89284**
Au	0.3	Pd	0.7	**-3.79427**	-3.77435	-3.83729	**-3.83796**
Au	0.4	Pd	0.6	-3.67871	**-3.68949**	**-3.77712**	-3.77613
Au	0.5	Pd	0.5	-3.58941	**-3.60635**	**-3.71764**	-3.71706
Au	0.6	Pd	0.4	**-3.5705**	-3.56939	-3.66274	**-3.66327**
Au	0.7	Pd	0.3	-3.5341	**-3.53871**	**-3.6106**	-3.61015
Au	0.8	Pd	0.2	-3.51218	**-3.51929**	-3.55816	**-3.55834**
Au	0.9	Pd	0.1	-3.48962	**-3.49046**	-3.50457	**-3.50459**

Table S9. Optimized AgPd NP CE starting from Janus and random initial structures using the NP and DM methods.

				NP method CE (eV/atom)		DM method CE (eV/atom)	
Metal i	Composition	Metal j	Composition	Janus	Random	Janus	Random
Ag	0.1	Pd	0.9	**-3.87438**	-3.87407	**-3.88268**	**-3.88268**
Ag	0.2	Pd	0.8	-3.75895	**-3.75898**	-3.77819	**-3.77854**
Ag	0.3	Pd	0.7	-3.62342	**-3.62721**	**-3.66342**	-3.66092
Ag	0.4	Pd	0.6	**-3.4885**	-3.48769	**-3.53924**	-3.53891
Ag	0.5	Pd	0.5	-3.35154	**-3.35572**	**-3.41482**	-3.41383
Ag	0.6	Pd	0.4	**-3.23528**	-3.23484	-3.29225	**-3.29266**
Ag	0.7	Pd	0.3	**-3.12559**	-3.12426	-3.16923	**-3.16991**
Ag	0.8	Pd	0.2	**-3.02309**	-3.02236	-3.04839	**-3.04844**
Ag	0.9	Pd	0.1	**-2.91813**	-2.91511	**-2.92794**	-2.92756

Table S10. Optimized PdPt NP CE starting from Janus and random initial structures using the NP and DM methods.

				NP method CE (eV/atom)		DM method CE (eV/atom)	
Metal i	Composition	Metal j	Composition	Janus	Random	Janus	Random
Pd	0.1	Pt	0.9	-5.712	**-5.71225**	**-5.73629**	**-5.73629**
Pd	0.2	Pt	0.8	**-5.53929**	-5.53818	-5.57671	**-5.57682**
Pd	0.3	Pt	0.7	-5.34173	**-5.34281**	-5.4085	**-5.40906**
Pd	0.4	Pt	0.6	**-5.13545**	-5.13487	-5.23242	**-5.23252**
Pd	0.5	Pt	0.5	-4.93091	**-4.93204**	**-5.04863**	-5.04852
Pd	0.6	Pt	0.4	-4.73554	**-4.73583**	**-4.85693**	-4.85667
Pd	0.7	Pt	0.3	-4.54247	**-4.54391**	-4.65419	**-4.65519**
Pd	0.8	Pt	0.2	-4.35878	**-4.35964**	**-4.44212**	-4.44186
Pd	0.9	Pt	0.1	-4.17103	**-4.17188**	**-4.21637**	-4.21635

Table S11. Optimized AgPt NP CE starting from Janus and random initial structures using the NP and DM methods.

				NP method CE (eV/atom)		DM method CE (eV/atom)	
Metal i	Composition	Metal j	Composition	Janus	Random	Janus	Random
Ag	0.1	Pt	0.9	-5.6489	**-5.64895**	-5.60609	**-5.6061**
Ag	0.2	Pt	0.8	**-5.39475**	-5.39427	-5.32055	**-5.32146**
Ag	0.3	Pt	0.7	-5.11498	**-5.11561**	-5.00094	**-5.00437**
Ag	0.4	Pt	0.6	**-4.82356**	-4.823	-4.669	**-4.67408**
Ag	0.5	Pt	0.5	-4.5153	**-4.51621**	**-4.33945**	-4.33158
Ag	0.6	Pt	0.4	-4.2015	**-4.20191**	**-4.03596**	-4.02469
Ag	0.7	Pt	0.3	-3.87139	**-3.8715**	**-3.72024**	-3.71873
Ag	0.8	Pt	0.2	**-3.52894**	-3.52871	**-3.41713**	-3.41581
Ag	0.9	Pt	0.1	**-3.17218**	-3.17214	-3.1145	**-3.11696**

Table S12. Optimized AuCu NP CE starting from Janus and random initial structures using the NP and DM methods.

				NP method CE (eV/atom)		DM method CE (eV/atom)	
Metal i	Composition	Metal j	Composition	Janus	Random	Janus	Random
Au	0.1	Cu	0.9	-3.72895	**-3.72896**	**-3.75161**	-3.75125
Au	0.2	Cu	0.8	**-3.70968**	-3.70965	**-3.74956**	-3.74836
Au	0.3	Cu	0.7	**-3.68647**	-3.6864	**-3.70426**	-3.69551
Au	0.4	Cu	0.6	-3.66104	**-3.66127**	-3.64074	**-3.64282**
Au	0.5	Cu	0.5	**-3.63312**	-3.63311	-3.59728	**-3.59821**
Au	0.6	Cu	0.4	-3.60266	**-3.60271**	-3.5725	**-3.57765**
Au	0.7	Cu	0.3	**-3.56939**	-3.56934	-3.55049	**-3.5569**
Au	0.8	Cu	0.2	-3.533	**-3.53307**	**-3.54239**	-3.53813
Au	0.9	Cu	0.1	**-3.49317**	-3.49315	-3.5036	**-3.50389**

Table S13. Optimized AuNi NP CE starting from Janus and random initial structures using the NP and DM methods.

				NP method CE (eV/atom)		DM method CE (eV/atom)	
Metal i	Composition	Metal j	Composition	Janus	Random	Janus	Random
Au	0.1	Ni	0.9	-4.83578	**-4.83706**	**-4.72832**	**-4.72832**
Au	0.2	Ni	0.8	-4.79609	**-4.79739**	-4.60563	**-4.60632**
Au	0.3	Ni	0.7	**-4.72372**	-4.72347	**-4.47135**	-4.4709
Au	0.4	Ni	0.6	-4.62575	**-4.62758**	**-4.3269**	-4.32619
Au	0.5	Ni	0.5	-4.50004	**-4.5024**	**-4.18031**	-4.17948
Au	0.6	Ni	0.4	**-4.35345**	-4.35046	**-4.03429**	-4.03412
Au	0.7	Ni	0.3	-4.17379	**-4.17524**	**-3.88798**	-3.88795
Au	0.8	Ni	0.2	-3.96759	**-3.96902**	**-3.74201**	-3.74182
Au	0.9	Ni	0.1	**-3.72632**	-3.72562	**-3.59608**	-3.59576

Table S14. Optimized CuPt NP CE starting from Janus and random initial structures using the NP and DM methods.

				NP method CE (eV/atom)		DM method CE (eV/atom)	
Metal i	Composition	Metal j	Composition	Janus	Random	Janus	Random
Cu	0.1	Pt	0.9	**-5.75977**	-5.7595	-5.7088	**-5.70883**
Cu	0.2	Pt	0.8	**-5.6172**	-5.61682	**-5.52539**	-5.52481
Cu	0.3	Pt	0.7	-5.44884	**-5.44953**	-5.32613	**-5.32638**
Cu	0.4	Pt	0.6	**-5.26391**	-5.2628	**-5.11969**	-5.11892
Cu	0.5	Pt	0.5	**-5.0581**	-5.05769	-4.90422	**-4.9046**
Cu	0.6	Pt	0.4	-4.83441	**-4.83764**	-4.68505	**-4.68529**
Cu	0.7	Pt	0.3	-4.59252	**-4.59389**	**-4.45943**	-4.45931
Cu	0.8	Pt	0.2	-4.33475	**-4.33553**	-4.22668	**-4.22716**
Cu	0.9	Pt	0.1	-4.05055	**-4.05096**	-3.98802	**-3.98808**

Table S15. Optimized PdNi NP CE starting from Janus and random initial structures using the NP and DM methods.

				NP method CE (eV/atom)		DM method CE (eV/atom)	
Metal i	Composition	Metal j	Composition	Janus	Random	Janus	Random
Pd	0.1	Ni	0.9	-4.8149	**-4.81517**	**-4.77128**	-4.7712
Pd	0.2	Ni	0.8	**-4.76999**	-4.76943	**-4.69497**	-4.69447
Pd	0.3	Ni	0.7	**-4.71033**	-4.70947	-4.60284	**-4.60322**
Pd	0.4	Ni	0.6	-4.63966	**-4.64043**	**-4.50464**	-4.5046
Pd	0.5	Ni	0.5	**-4.55946**	-4.55921	-4.40875	**-4.41188**
Pd	0.6	Ni	0.4	**-4.46898**	-4.46879	**-4.32054**	-4.3204
Pd	0.7	Ni	0.3	**-4.36627**	-4.36537	**-4.23411**	-4.23406
Pd	0.8	Ni	0.2	-4.25236	**-4.25285**	**-4.15268**	-4.152
Pd	0.9	Ni	0.1	**-4.12412**	-4.12389	**-4.06754**	-4.06719

Table S16. Optimized AuAg NP CE starting from Janus and random initial structures using the NP and DM methods.

				NP method CE (eV/atom)		DM method CE (eV/atom)	
Metal i	Composition	Metal j	Composition	Janus	Random	Janus	Random
Au	0.1	Ag	0.9	**-2.87**	-2.86926	**-2.90854**	-2.90844
Au	0.2	Ag	0.8	**-2.93349**	-2.93279	-3.00378	**-3.00433**
Au	0.3	Ag	0.7	**-2.9959**	-2.99431	**-3.0898**	-3.08969
Au	0.4	Ag	0.6	**-3.05986**	-3.05952	-3.1676	**-3.16785**
Au	0.5	Ag	0.5	**-3.12621**	-3.12577	-3.23552	**-3.23585**
Au	0.6	Ag	0.4	-3.19757	**-3.19761**	-3.29809	**-3.29897**
Au	0.7	Ag	0.3	-3.26855	**-3.26916**	**-3.35301**	-3.35286
Au	0.8	Ag	0.2	**-3.33792**	-3.33767	**-3.39756**	-3.3973
Au	0.9	Ag	0.1	-3.39543	**-3.39544**	-3.43138	**-3.4314**

Table S17. Optimized AgNi NP CE starting from Janus and random initial structures using the NP and DM methods.

				NP method CE (eV/atom)		DM method CE (eV/atom)	
Metal i	Composition	Metal j	Composition	Janus	Random	Janus	Random
Ag	0.1	Ni	0.9	**-4.7544**	-4.75424	-4.66022	**-4.66053**
Ag	0.2	Ni	0.8	-4.64441	**-4.64443**	-4.4664	**-4.46879**
Ag	0.3	Ni	0.7	-4.50705	**-4.5085**	-4.24889	**-4.25437**
Ag	0.4	Ni	0.6	**-4.34466**	-4.3428	-4.02001	**-4.03144**
Ag	0.5	Ni	0.5	**-4.15717**	-4.15714	**-3.80662**	-3.80543
Ag	0.6	Ni	0.4	**-3.94701**	-3.94485	**-3.59691**	-3.59255
Ag	0.7	Ni	0.3	-3.7077	**-3.7092**	-3.39795	**-3.39986**
Ag	0.8	Ni	0.2	-3.44265	**-3.4446**	**-3.20631**	-3.20159
Ag	0.9	Ni	0.1	**-3.13965**	-3.13949	-3.00455	**-3.0065**

Table S18. Optimized CuNi NP CE starting from Janus and random initial structures using the NP and DM methods.

				NP method CE (eV/atom)		DM method CE (eV/atom)	
Metal i	Composition	Metal j	Composition	Janus	Random	Janus	Random
Cu	0.1	Ni	0.9	**-4.84346**	-4.84336	**-4.74906**	-4.74899
Cu	0.2	Ni	0.8	**-4.81138**	-4.81135	-4.64665	**-4.64751**
Cu	0.3	Ni	0.7	**-4.75122**	-4.75037	-4.53282	**-4.53312**
Cu	0.4	Ni	0.6	**-4.66887**	-4.66652	-4.40661	**-4.40998**
Cu	0.5	Ni	0.5	**-4.56306**	-4.5627	-4.2852	**-4.28604**
Cu	0.6	Ni	0.4	-4.44364	**-4.44505**	-4.17307	**-4.17383**
Cu	0.7	Ni	0.3	-4.30229	**-4.3023**	-4.06462	**-4.06482**
Cu	0.8	Ni	0.2	-4.13859	**-4.13888**	**-3.96119**	-3.96096
Cu	0.9	Ni	0.1	**-3.95051**	-3.95043	-3.85337	**-3.85348**

3. Chemical ordering in 561- and 2869- atom AuAg NPs

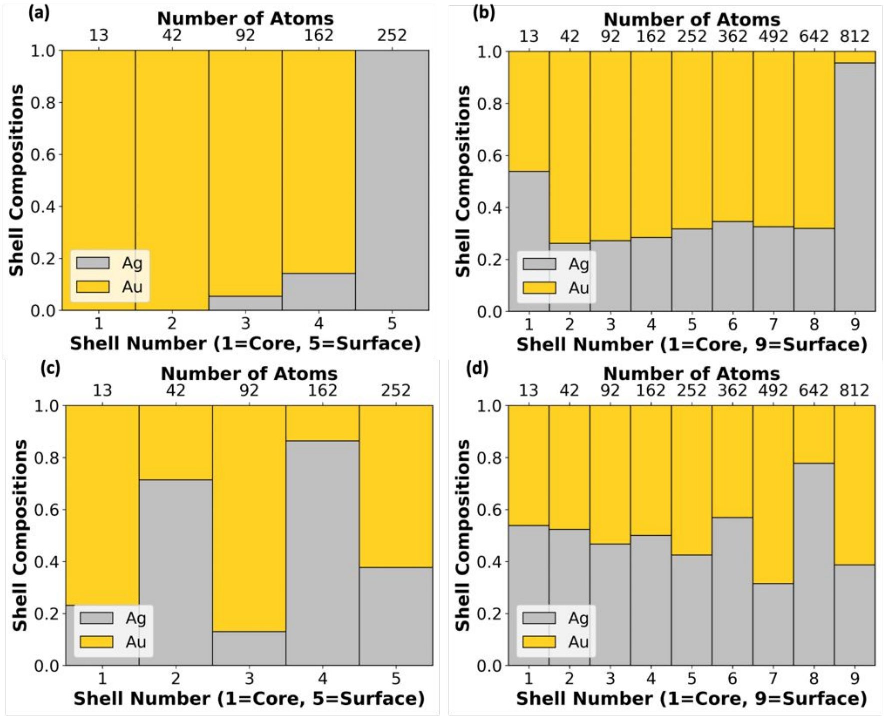

Figure S1. Core to shell metal distribution of AuAg obtained using the NP method for (a) 561 and (b) 2869-atom NP and the DM method for (c) 561 and (d) 2869-atom NP, respectively.

4. Agreement in the chemical ordering between the DM and NP Methods

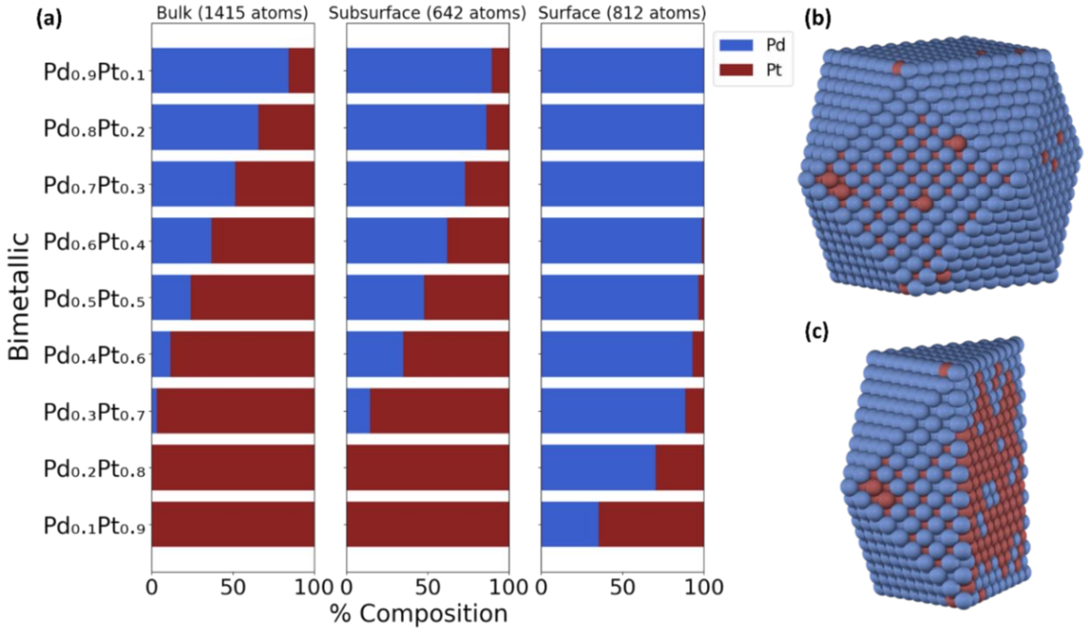

Figure S2. Chemical ordering of (a) 2869 atom-PdPt cuboctahedron NP as a function of bulk, subsurface and surface composition using the NP method and a NP with 50/50 composition with its corresponding (b) surface and (c) center-cut projection.

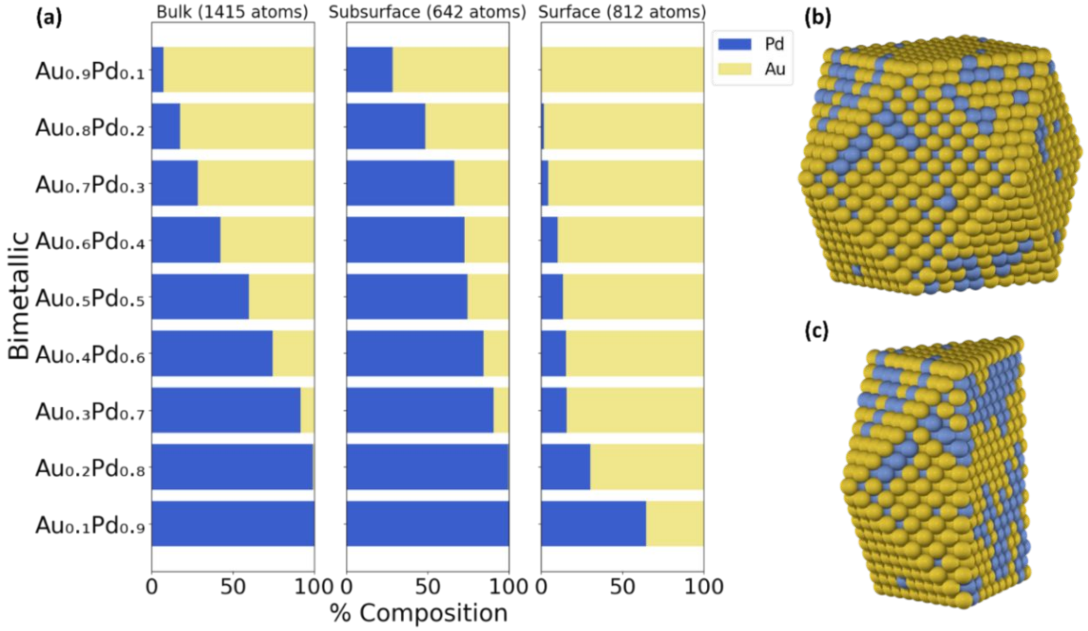

Figure S3. Chemical ordering of (a) 2869 atom-AuPd cuboctahedron NP as a function of bulk, subsurface and surface composition using the NP method and a NP with 50/50 composition with its corresponding (b) surface and (c) center-cut projection.

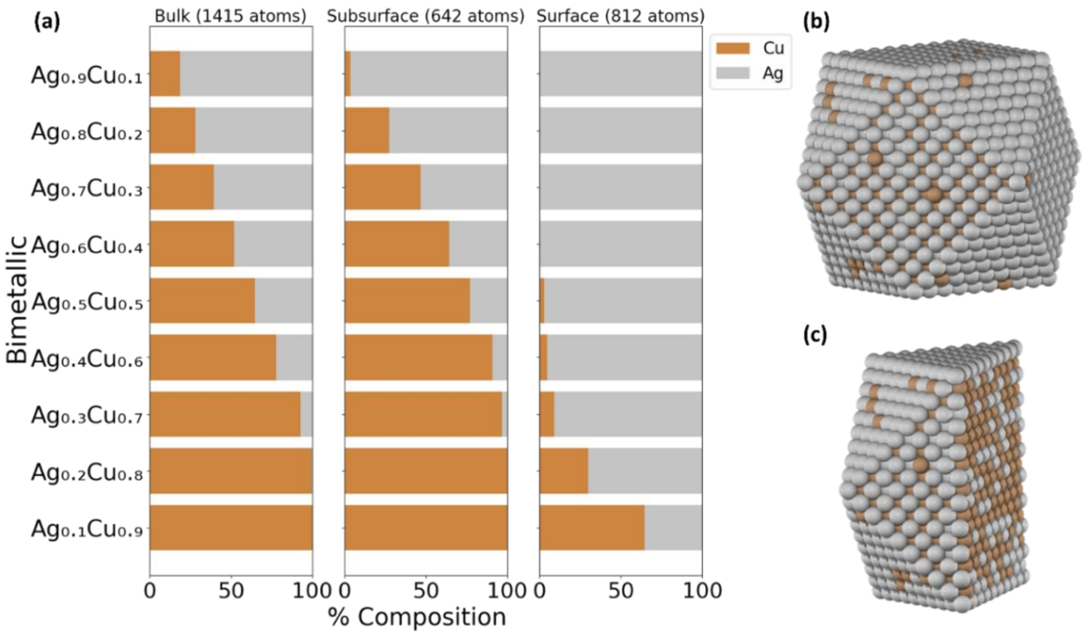

Figure S4. Chemical ordering of (a) 2869 atom-AgCu cuboctahedron NP as a function of bulk, subsurface and surface composition using the NP method and a NP with 50/50 composition with its corresponding (b) surface and (c) center-cut projection.

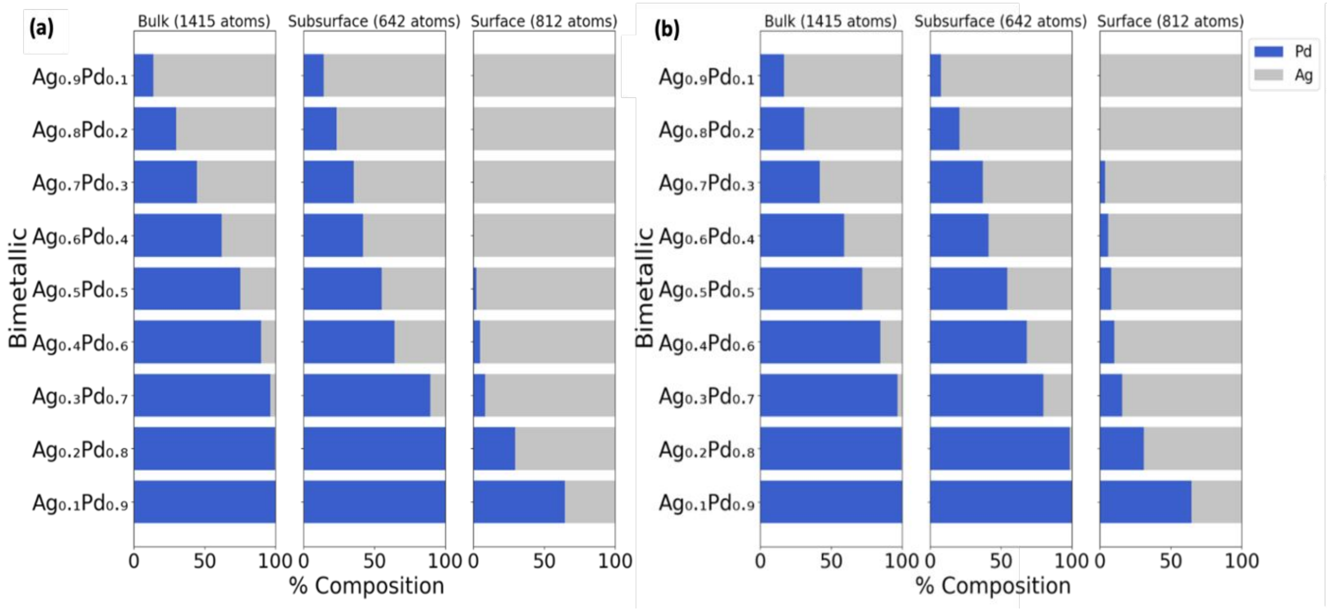

Figure S5. Chemical ordering of 2869 atom-AgPd cuboctahedron NP as a function of bulk, subsurface and surface composition using (a) the DM and (b) NP method.

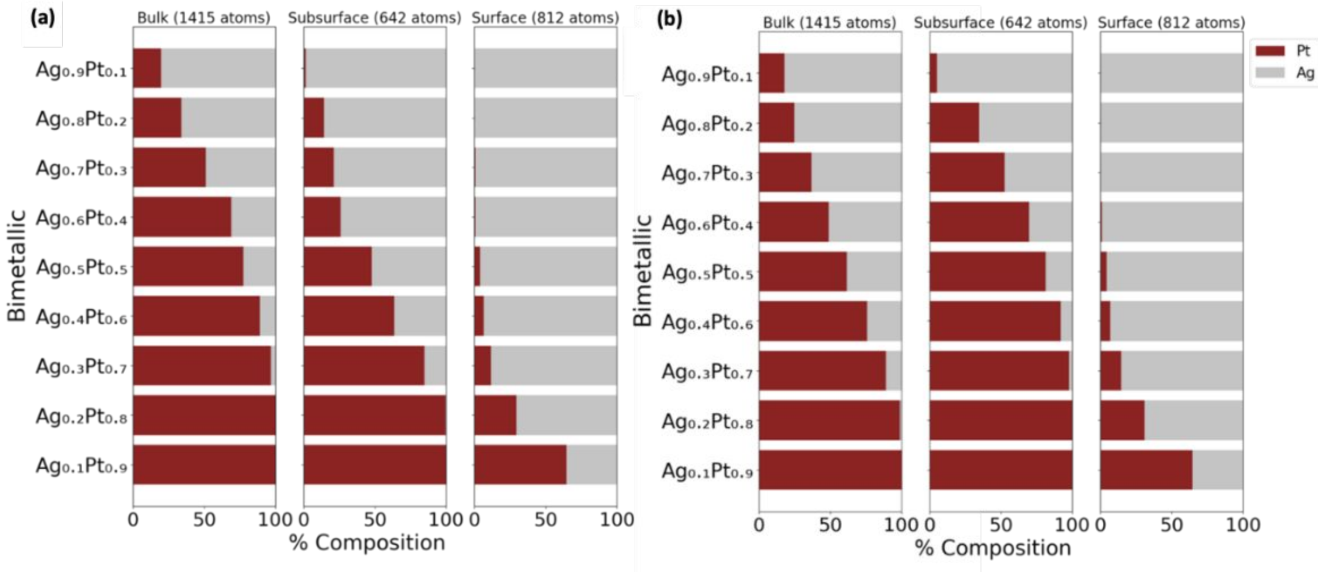

Figure S6. Chemical ordering of 2869 atom-AgPt cuboctahedron NP as a function of bulk, subsurface and surface composition using (a) the DM and (b) NP method.

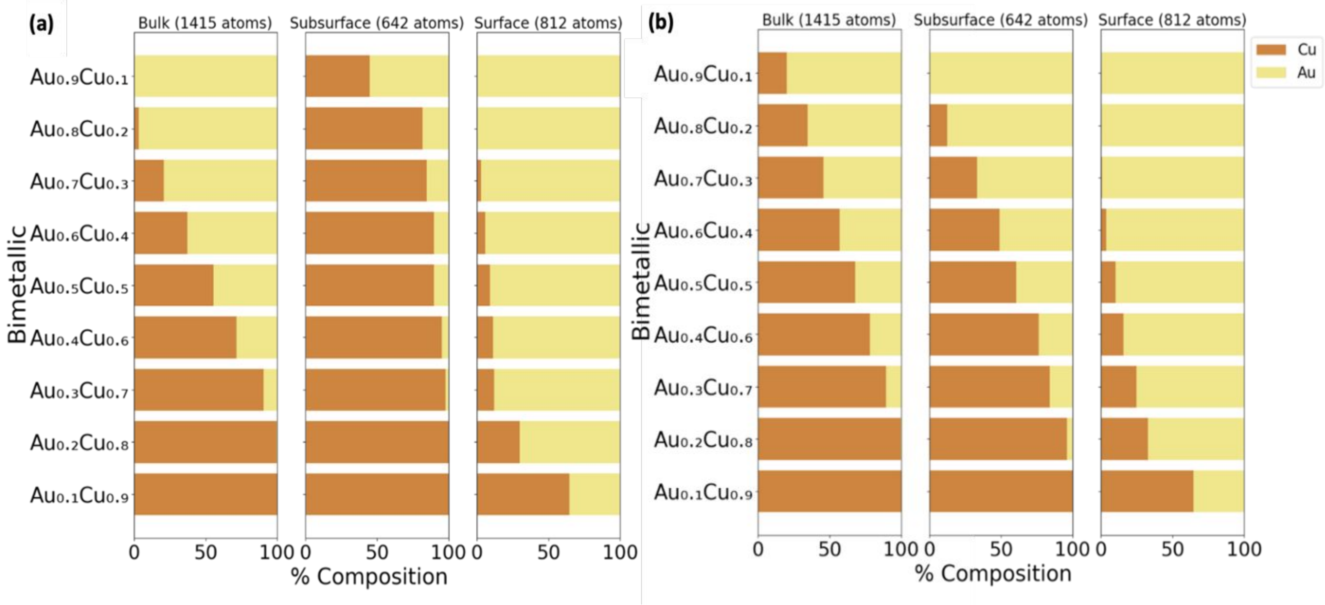

Figure S7. Chemical ordering of 2869 atom-AuCu cuboctahedron NP as a function of bulk, subsurface and surface composition using (a) the DM and (b) NP method.

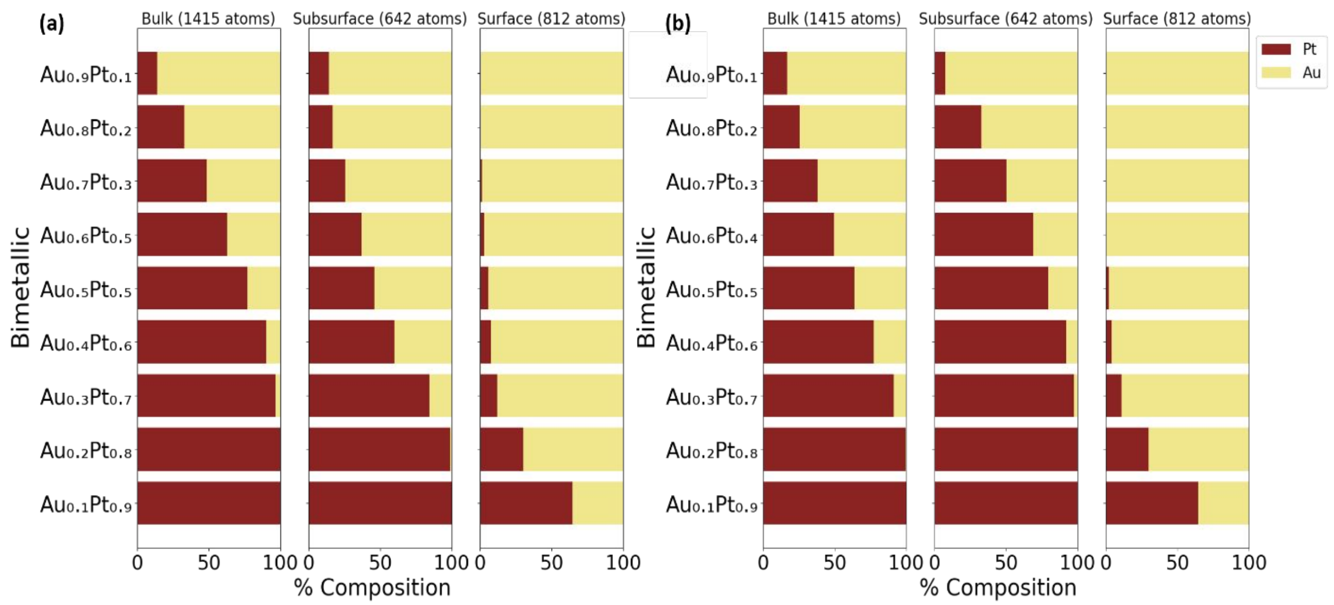

Figure S8. Chemical ordering of 2869 atom-AuPt cuboctahedron NP as a function of bulk, subsurface and surface composition using (a) the DM and (b) NP method.

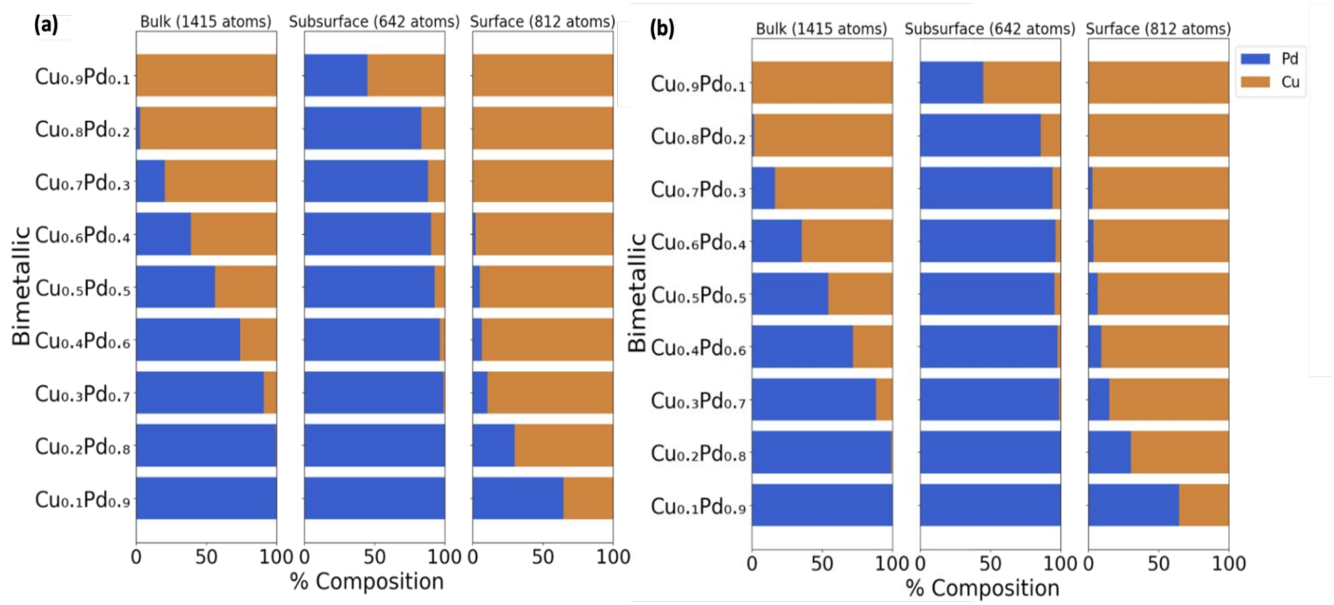

Figure S9. Chemical ordering of 2869 atom-CuPd cuboctahedron NP as a function of bulk, subsurface and surface composition using (a) the DM and (b) NP method.

Platinum-Nickel

It was revealed that for a NP with a composition of $Pt_{0.4}Ni_{0.6}$, the surface of the (100) plane is characterized as Pt-rich, indicating that platinum atoms are more prevalent compared to Ni; in contrast, the (111) plane features a Ni-rich outermost layer, suggesting that nickel atoms dominate the surface composition in this plane[1] (similar to our predictions depicted in Table S19). Also, the inner part of the nanoparticle displays a stable Ni-rich composition[1], which matches our $Pt_{0.4}Ni_{0.6}$ NP shown in Figure S10 for the DM method.

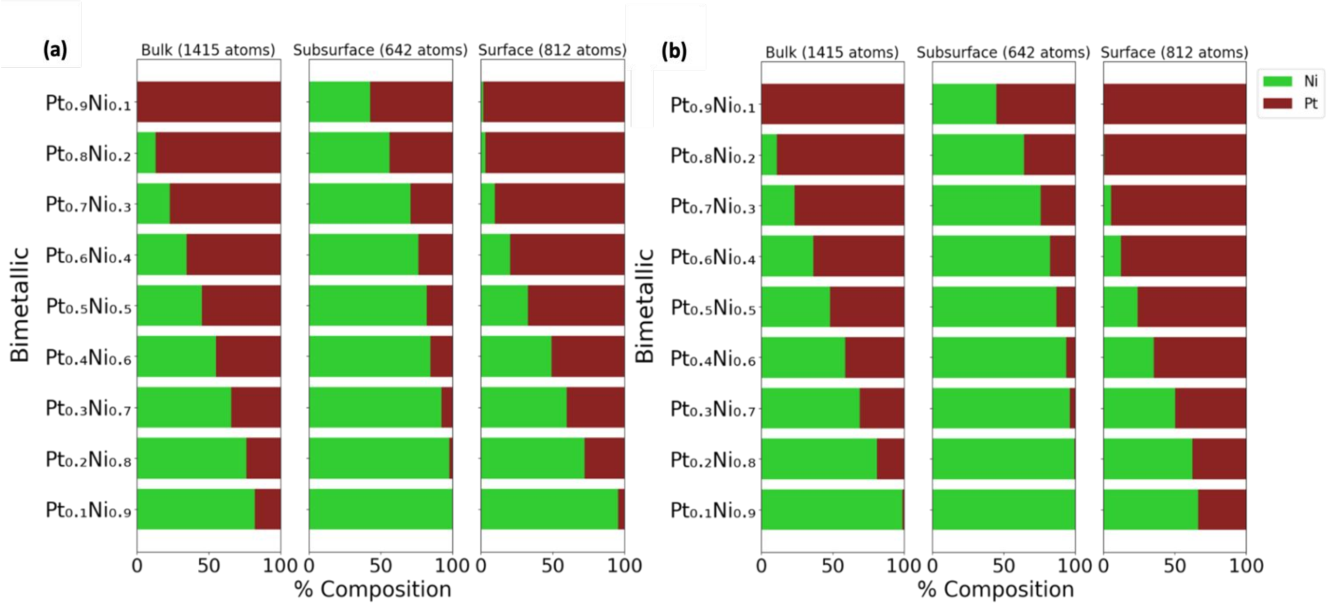

Figure S10. Chemical ordering of 2869 atom-PtNi cuboctahedron NP as a function of bulk, subsurface and surface composition using (a) the DM method and (b) NP method.

Table S19. Percentage of each metal on (100) and (111) surfaces relative to the total surface of the NP using the DM method

	**(100) plane**		**(111) plane**	
**Composition**	**Pt**	**Ni**	**Pt**	**Ni**
$Pt_{0.1}Ni_{0.9}$	1.254793	12.12966	0	7.807598
$Pt_{0.2}Ni_{0.8}$	4.984315	8.400139	0.906239	6.901359
$Pt_{0.3}Ni_{0.7}$	6.517951	6.866504	1.882189	5.92541
$Pt_{0.4}Ni_{0.6}$	7.94702	5.437435	2.649007	5.158592
$Pt_{0.5}Ni_{0.5}$	10.70059	2.683862	3.346114	4.461485
$Pt_{0.6}Ni_{0.4}$	12.4085	0.97595	4.252353	3.555246
$Pt_{0.7}Ni_{0.3}$	13.17532	0.209132	5.437435	2.370164
$Pt_{0.8}Ni_{0.2}$	13.38445	0	6.97107	0.836528
$Pt_{0.9}Ni_{0.1}$	13.38445	0	7.389334	0.418264

The values in the table represent the percentage of each metal on the (100) and (111) surfaces relative to the total number of surface atoms in the NP. For example, in the $Pt_{0.1}Ni_{0.9}$ composition (where the NP contains 10% Pt overall), the table shows that only 1.25% of the total surface atoms are Pt on the (100) facet. This means that Pt is not evenly distributed across all surface facets. Additionally, the NP surface is not exclusively composed of (100) and (111) facets; other orientations and coordination environments (e.g., CN=7, CN=6) also contribute. This is why the percentages in the table do not sum to 100%.

5. Discrepancy in the chemical ordering between the DM and NP Methods

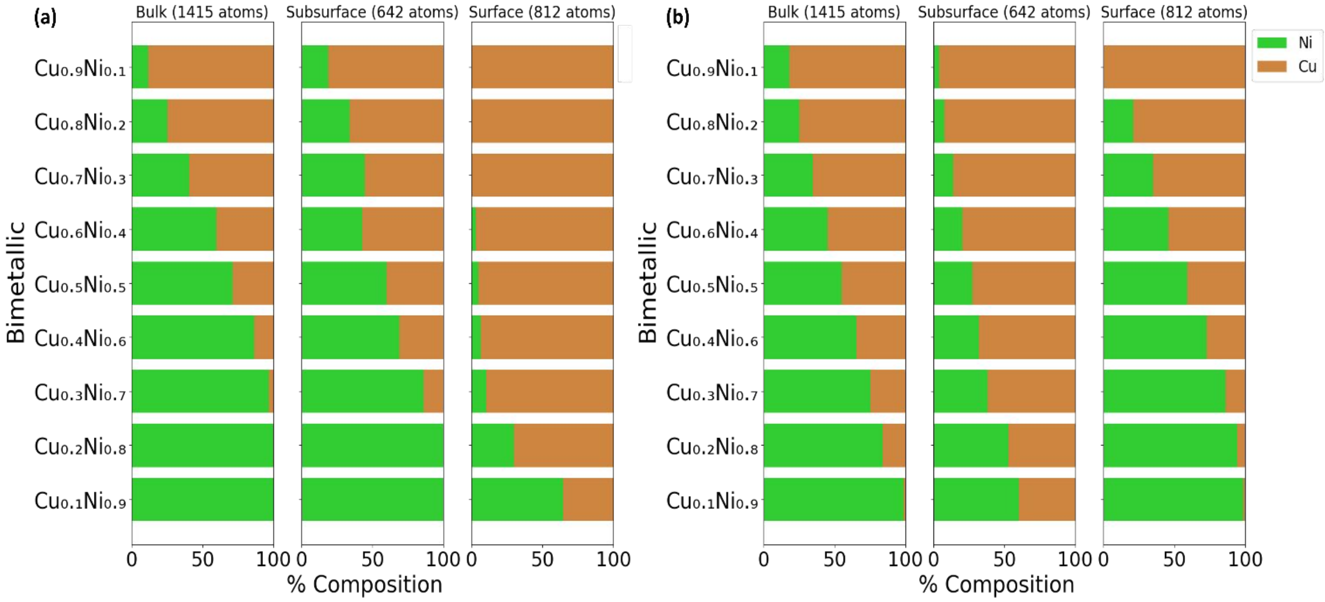

Figure S11. Chemical ordering of 2869 atom-CuNi cuboctahedron NP as a function of bulk, subsurface and surface composition using the (a) DM and (b) NP method.

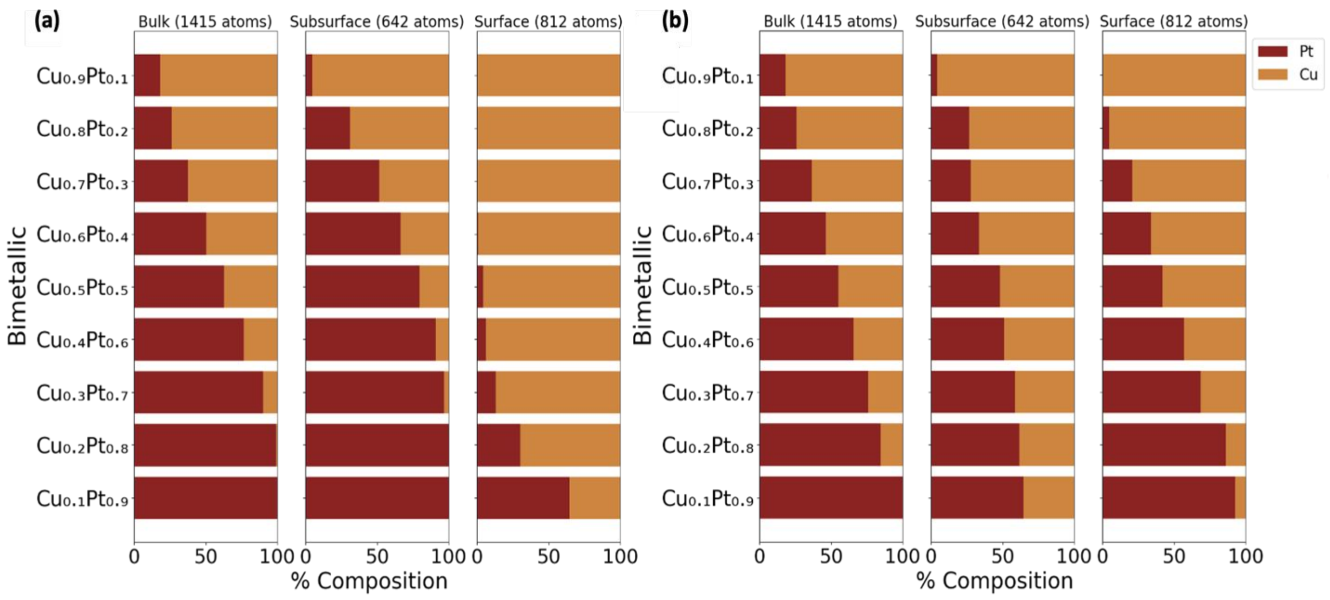

Figure S12. Chemical ordering of 2869 atom-PtCu cuboctahedron NP as a function of bulk, subsurface and surface composition using the (a) DM and (b) NP method.

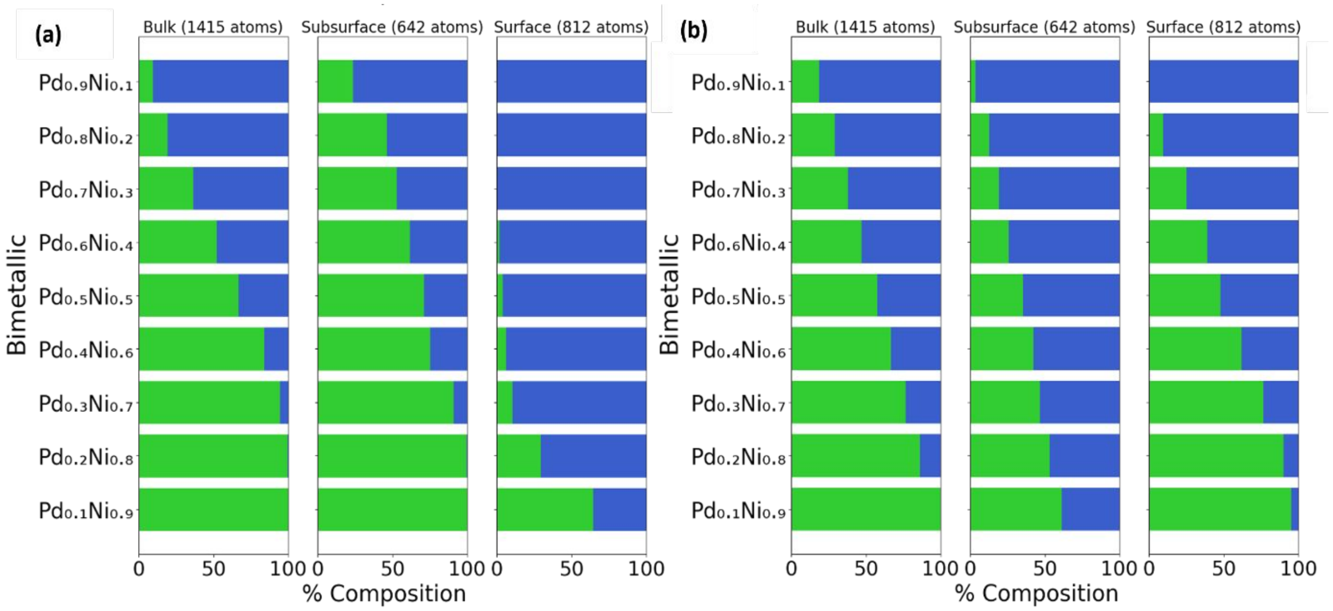

Figure S13. Chemical ordering of 2869 atom-PdNi cuboctahedron NP as a function of bulk, subsurface and surface composition using the (a) DM and (b) NP method.

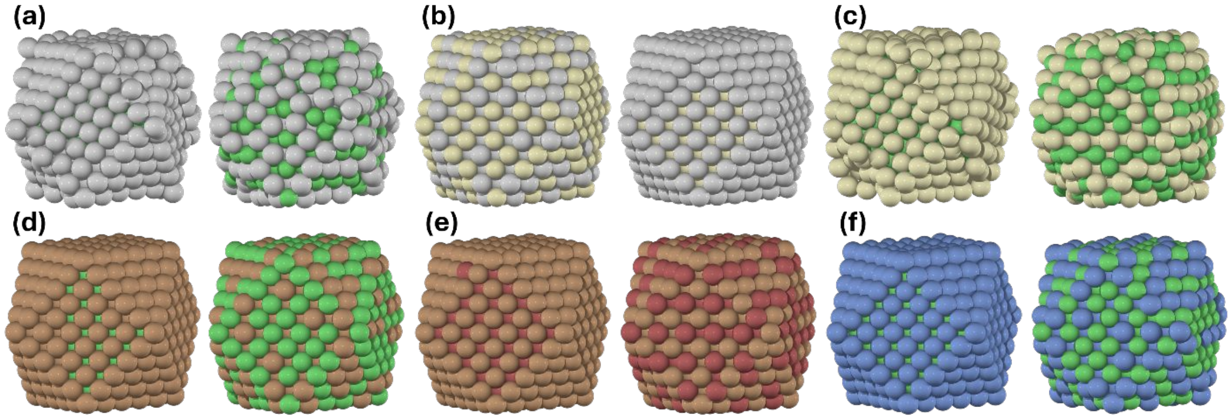

Figure S14. DFT-optimized structures of 561-atom nanoparticles (NPs) with 50/50 composition for (a) AgNi, (b) AuAg, (c) AuNi, (d) CuNi, (e) PtCu, and (f) PdN. For each NP, GA- and BCM-derived structures are shown, with DM-optimized configurations on the left and NP-optimized configurations on the right.

6. CE_{bulk}, DFT and computational and experimental analysis

Table S20. CE_{bulk}, DFT and literature results for combinations that show discrepancy in the chemical ordering for DM and NP methods

Metals	Diff in CE_{bulk} (in eV)	NP Surface Based on the CE_{bulk}	NP method Surface Predictions	DM method Surface Predictions	NP Surface Based on Experimental/ Computational Observations	DFT CE Results (which is more negative?)
AuAg	0.68	Ag	Ag	61%Au	**61%Au**[33–35]	DM
AuNi	-1.47	Au	51%Au	Au	**Au**[2,3]	DM
AgNi	-2.15	Ag	65%Ag	Ag	**Ag**[4,5]	DM
NiCu	1.16	Cu	59%Ni	Cu	**Cu**[6,7]	DM
NiPd	0.91	Pd	48%Ni	Pd	**Pd**[8–10]	DM
PtCu	2.25	Cu	42%Pt	Cu	**Cu**[11,12]	NP (≈0.0067 eV/atom)

7. DM method results for trimetallic NPs

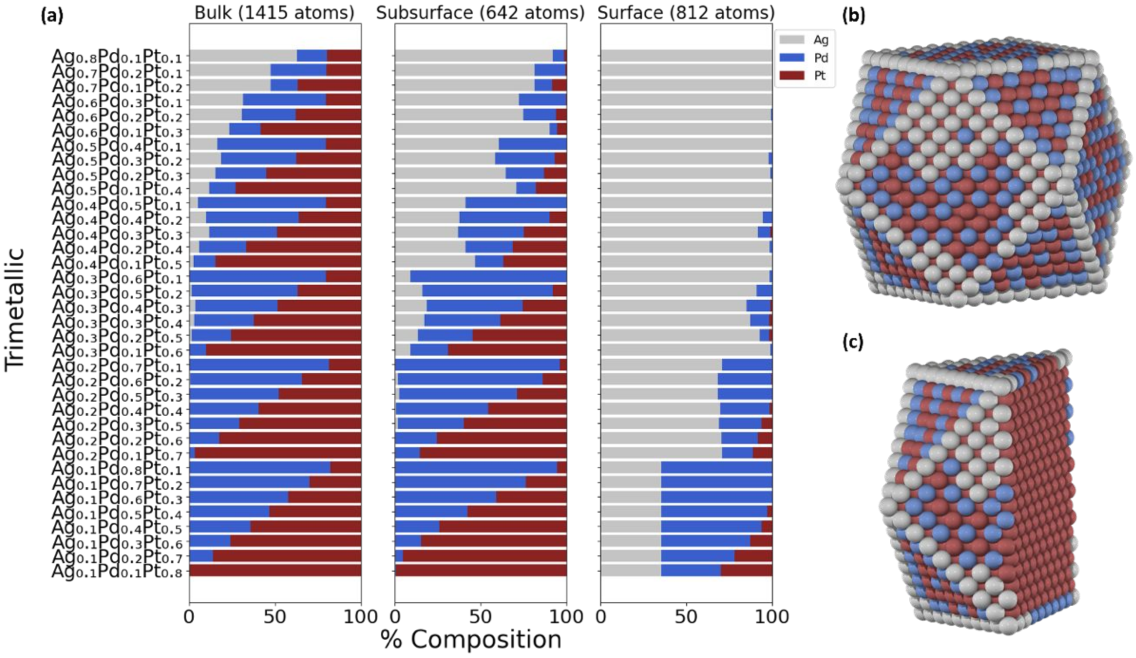

Figure S15. Chemical ordering of (a) 2869 atom-AgPdPt cuboctahedron NP as a function of bulk, subsurface and surface composition using the DM method, and a NP with 10/10/80 composition with its corresponding (b) surface and (c) center-cut projection.

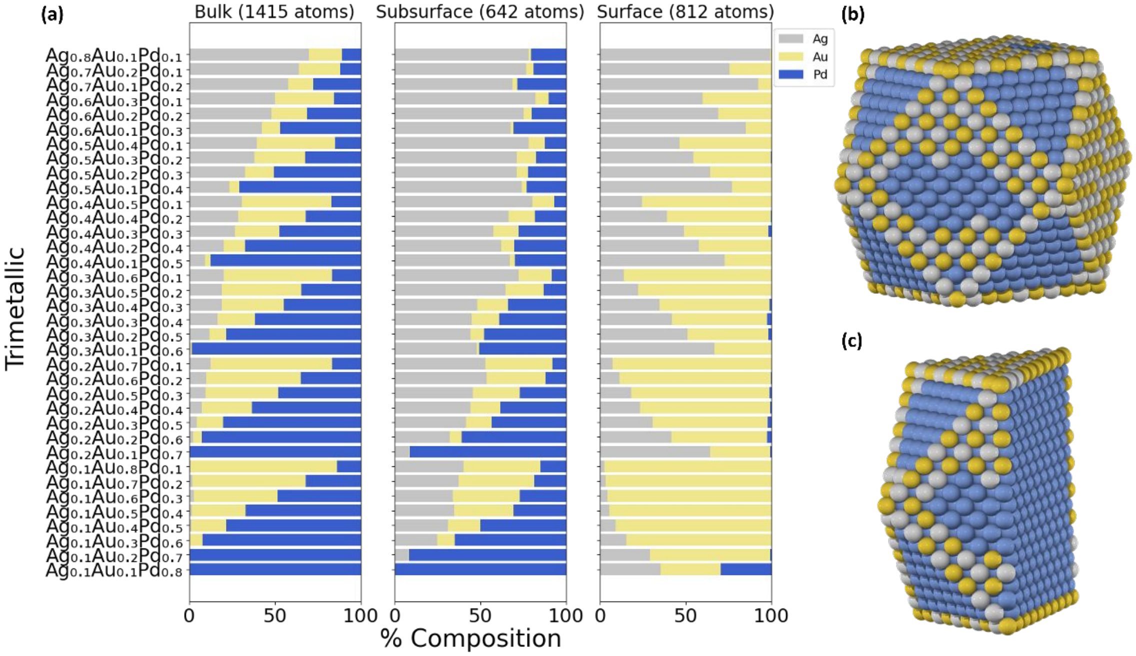

Figure S16. Chemical ordering of (a) 2869 atom-AuAgPd cuboctahedron NP as a function of bulk, subsurface and surface composition using the DM method and a NP with 10/10/80 composition with its corresponding (b) surface and (c) center-cut projection.

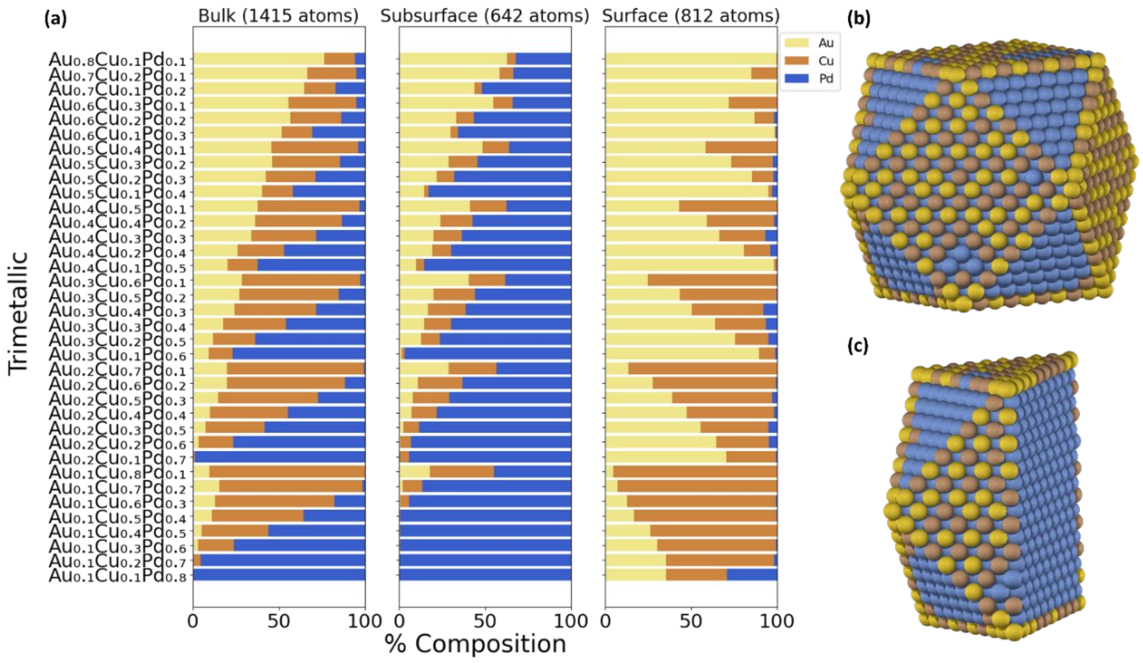

Figure S17. Chemical ordering of (a) 2869 atom-AuCuPd cuboctahedron NP as a function of bulk, subsurface and surface composition using the DM method and a NP with 10/10/80 composition with its corresponding (b) surface and (c) center-cut projection.

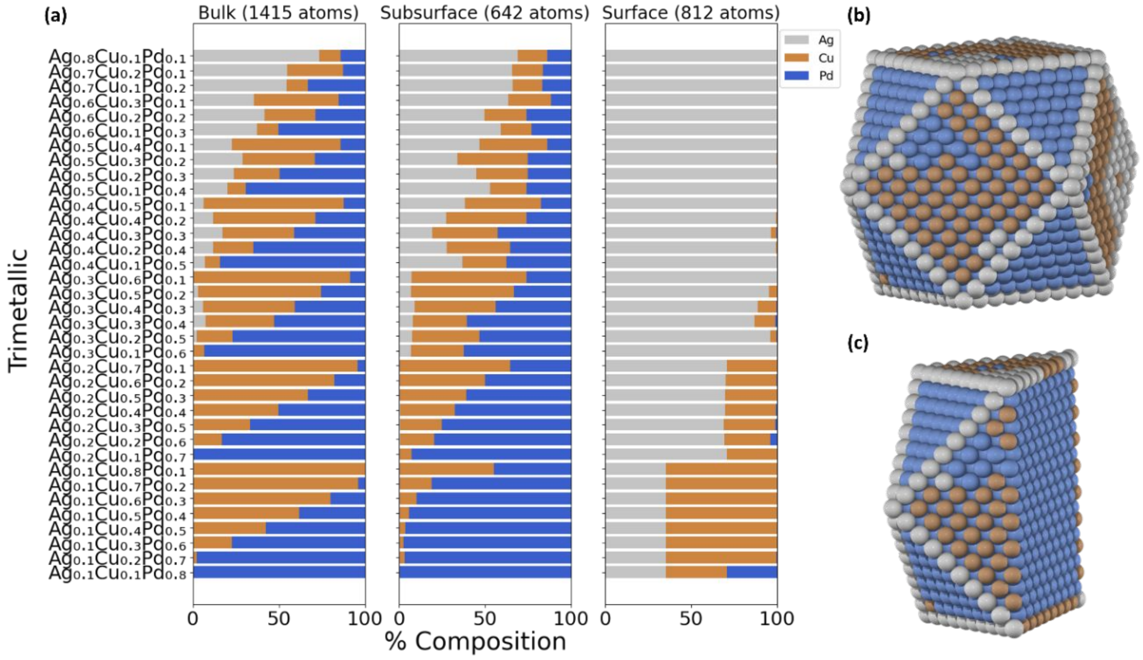

Figure S18. Chemical ordering of (a) 2869 atom-AgCuPd cuboctahedron NP as a function of bulk, subsurface and surface composition using the DM method, and a NP with 10/10/80 composition with its corresponding (b) surface and (c) center-cut projection.

References

1. Li, Q. *et al.* Local Chemical Ordering and Negative Thermal Expansion in PtNi Alloy Nanoparticles. *Nano Lett* **17**, 7892–7896 (2017).

2. Bogatyrenko, S., Kryshtal, A., Minenkov, A. & Kruk, A. Miscibility gap narrowing on the phase diagram of Au Ni nanoparticles. *Scr Mater* **170**, 57–61 (2019).

3. Rousset, J. L. *et al.* Comparative X-ray Photoemission Spectroscopy Study of Au, Ni, and AuNi Clusters Produced by Laser Vaporization of Bulk Metals. *J Phys Chem B* **104**, 5430–5435 (2000).

4. Li, M. M.-J. *et al.* Surfactant-free nickel–silver core@shell nanoparticles in mesoporous SBA-15 for chemoselective hydrogenation of dimethyl oxalate. *Chemical Communications* **52**, 2569–2572 (2016).

5. Cottancin, E. *et al.* Optical properties of mixed clusters: comparative study of Ni/Ag and Pt/Ag clusters. *Eur Phys J D At Mol Opt Phys* **24**, 111–114 (2003).

6. Helms, C. R. & Yu, K. Y. Determination of the surface composition of the Cu−Ni alloys for clean and adsorbate−covered surfaces. *Journal of Vacuum Science and Technology* **12**, 276–278 (1975).

7. Wang, Q., Wang, X., Liu, J. & Yang, Y. Cu–Ni core–shell nanoparticles: structure, stability, electronic, and magnetic properties: a spin-polarized density functional study. *Journal of Nanoparticle Research* **19**, 25 (2017).

8. Derry, G. N., Wan, R., Krueger, E., Waldt, J. & English, C. Structure and composition of the NiPd(110) surface. *Surf Sci* **603**, 2193–2199 (2009).

9. Derry, G. N., McVey, C. B. & Rous, P. J. The surface structure and segregation profile of Ni50Pd50(100): a dynamical LEED study. *Surf Sci* **326**, 59–66 (1995).

10. Derry, G. N., Wan, R., Strauch, F. & English, C. Segregation and interlayer relaxation at the NiPd(111) surface. *Journal of Vacuum Science & Technology A: Vacuum, Surfaces, and Films* **29**, (2011).

11. Spadaro, M. C. *et al.* Electrocatalytic Behavior of PtCu Clusters Produced by Nanoparticle Beam Deposition. *The Journal of Physical Chemistry C* **124**, 23683–23689 (2020).

12. Yun, K. *et al.* Monte Carlo simulations of the structure of Pt-based bimetallic nanoparticles. *Acta Mater* **60**, 4908–4916 (2012).
